# Nanoarchitectonics of copper sulfide nanoplating for improvement of computed tomography efficacy of bismuth oxide constructs toward drugless theranostics

**DOI:** 10.1093/rb/rbae128

**Published:** 2024-10-26

**Authors:** Ruo-Yin Meng, Hong-Ying Xia, Ying Zhao, Ying-Tong Ye, Shi-Bin Wang, Ai-Zheng Chen, Ranjith Kumar Kankala

**Affiliations:** Institute of Biomaterials and Tissue Engineering, Huaqiao University, Xiamen 361021, PR China; College of Chemical Engineering, Huaqiao University, Xiamen 361021, PR China; Institute of Biomaterials and Tissue Engineering, Huaqiao University, Xiamen 361021, PR China; College of Chemical Engineering, Huaqiao University, Xiamen 361021, PR China; Institute of Biomaterials and Tissue Engineering, Huaqiao University, Xiamen 361021, PR China; College of Chemical Engineering, Huaqiao University, Xiamen 361021, PR China; Institute of Biomaterials and Tissue Engineering, Huaqiao University, Xiamen 361021, PR China; College of Chemical Engineering, Huaqiao University, Xiamen 361021, PR China; Institute of Biomaterials and Tissue Engineering, Huaqiao University, Xiamen 361021, PR China; College of Chemical Engineering, Huaqiao University, Xiamen 361021, PR China; Fujian Provincial Key Laboratory of Biochemical Technology, Huaqiao University, Xiamen 361021, PR China; Institute of Biomaterials and Tissue Engineering, Huaqiao University, Xiamen 361021, PR China; College of Chemical Engineering, Huaqiao University, Xiamen 361021, PR China; Fujian Provincial Key Laboratory of Biochemical Technology, Huaqiao University, Xiamen 361021, PR China; Institute of Biomaterials and Tissue Engineering, Huaqiao University, Xiamen 361021, PR China; College of Chemical Engineering, Huaqiao University, Xiamen 361021, PR China; Fujian Provincial Key Laboratory of Biochemical Technology, Huaqiao University, Xiamen 361021, PR China

**Keywords:** computed tomography, photothermal therapy, chemodynamic therapy, core-shell architectures, copper sulfide, bismuth oxide

## Abstract

Triple-negative breast cancer (TNBC) has emerged as one of the dreadful metastatic tumors in women due to complexity, specificity and high recurrence, resulting in poor therapeutic outcomes and requiring real-time monitoring for improved theranostics. Despite the success as efficient radiosensitizers and computed tomography (CT)-based contrast agents, bismuth (Bi)-based composites suffer from poor colloidal stability, dose-dependent toxicity and pharmacokinetic shortcomings, leading to poor therapeutic monitoring. In addition, several small molecule-based therapeutics, including nanoparticle-based delivery systems, suffer from several limitations of poor therapeutic delivery and acquired multidrug resistance by cancer cells, depriving the therapeutic needs. To overcome this aspect, this study demonstrates the fabrication of drug-like/drugless nanoarchitectures based on copper sulfide-nanoplated bismuth oxide (Bi_2_O_3_@CuS, shortly BC) composites for improved theranostic efficacy against TNBC. These systematically characterized BC nanocomposites exhibited pH-/near-infrared (NIR, 808 nm) light-responsive degradability toward dual modal therapies. Due to the band transition of Cu species, the designed BC composites displayed exceptional photothermal (PTT) conversion efficiency toward localized PTT effects. In addition to pH-/NIR-responsiveness, the internally overexpressed glutathione (GSH)-responsiveness facilitated the release of Cu^2+^ species for chemodynamic therapy (CDT)-based effects. To this end, the Bi^3+^ species in the core could be fully hydrated in the acidic tumor microenvironment, resulting in GSH depletion and reducing CDT-induced reactive oxygen species clearance, thereby ablating tumors. The acid-responsive degradability of CuS resulted in the intratumoral enrichment of BC, demonstrating remarkable CT imaging efficacy *in vivo*. Together, these pH-/NIR-/GSH-responsive biodegradable BC composites could realize the integrated PTT/CDT/CT theranostics against breast carcinoma.

## Graphical abstract

**Figure:**
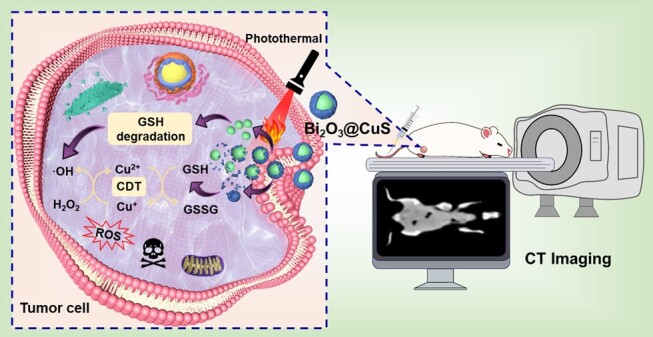

## Introduction

Cancer has emerged as one of the major global healthcare challenges, i.e. the second most affected ailment after cardiovascular diseases, accounting for millions of new cases annually [1–3]. Despite the availability of several treatment modalities, the eventual application of chemotherapeutic regimens using small-molecule therapeutics is inevitable to avoid recurrence [4–7]. Nevertheless, various small-molecule-based therapeutics often suffer from several limitations, such as poor bioavailability due to hydrophobicity, serious adverse effects on normal cells due to poor targeting efficacy, and acquired multidrug resistance, leading to reduced therapeutic efficiency [8–11]. According to cancer data statistics in 2022 [12], triple-negative breast cancer (TNBC) has emerged as one of the most serious phenotypes of breast cancer, mainly characterized by local recurrence and distant metastasis due to poor treatment prognosis [13], leading to difficulties in treatment and poor overall survival rate [14]. Despite the exploration of diverse treatment modalities [15], it is important to explore some intelligent materials, such as drug-like/drugless architectures, to design selective therapeutic strategies for real-time monitoring against TNBC [16].

Among several stimuli, including pH [17], light [18], molecular [19] and magnetic [20], among others [21], light-based photothermal-based therapy (PTT) has emerged as one of the most important non-invasive strategies that use various active photothermal conversion agents (PTCAs) [22], converting irradiated light (specifically, near-infrared, NIR region) energy into heat toward selective ablation of tumor cells [23, 24]. These PTCAs with a high absorption in the NIR region, especially at 808 nm, result in an enriched localized temperature without affecting the surrounding tissues [25]. In recent times, various kinds of inorganic nanoassemblies have been increasingly recognized as photosensitizers to act against multiple tumors [26–30]. Copper sulfide (CuS)-based nanoconstructs offer numerous intrinsic characteristics, such as exceptional photothermal conversion efficiency, ease of synthesis, excellent biocompatibility and biodegradability *in vivo*, among others [31]. Over a decade, several efforts have been dedicated to fabricating CuS-based constructs to replace gold nanoparticles as PTCAs due to the d-d band transition of Cu^2+^ ions in the NIR region (700–1100 nm) and the unaffected absorption in the physiological environment [32]. In addition, copper ions participate in the Fenton-like reaction under a weakly acidic environment, converting intracellular hydrogen peroxide to toxic hydroxyl (•OH) radicals in the tumor microenvironment (TME) to induce chemodynamic therapy (CDT) [33]. Recent studies on CuS nanomaterials indicated that hyperthermia induced by laser irradiation could promote Fenton-like reaction, accelerating the reactive oxygen species (ROS) generation at tumor sites and enabling nanomaterial-mediated PTT and CDT effects synergistically against tumors [34–36]. However, CuS-based nanoconstructs suffer from a major limitation of dose-dependent toxicity to normal cells, resulting in their inability to monitor the real-time treatment process [37–39]. Owing to these issues related to cancer therapy, it is of particular importance to explore intelligent materials with exceptional theranostic modalities.

Considerably, different materials for bioimaging combined with PTT agents have become superior interest in developing versatile intelligent nanosystems [40–42], such as manganese dioxide (MnO_2_) for magnetic resonance imaging (MRI) [43], Bismuth (Bi) for X-ray computed tomography (CT) imaging [44], and gold (Au) for photoacoustic (PA) imaging [45]. CT imaging offers the major advantage of providing a 3D view with real-time feedback on the altered tissue lesions [46]. Notably, elements with a high atomic number can produce high-energy photoelectrons and low-energy Auger electrons, effectively hydrolyzing the surrounding water molecules [47]. As a member of a high atomic number element, Bi (*Z* = 83) exhibits specific physicochemical properties, relative stability and significantly high X-ray attenuation coefficient toward integrated CT imaging performance attributes (Bi: 5.74 cm^2^/g, Au: 5.16 cm^2^/g) [48–50]. Considerably, several conventional Bi-based oxides and sulfides offer pH-responsiveness at low pH values, degrading into Bi ions in TME (pH = 6.4–7.2) and excreted through the kidneys with minimal impact [51, 52]. Bi-based oxides (specifically, Bi_2_O_3_) are preferred over Bi-based sulfides due to their lower toxicity, convenience to synthesize, higher stability and ideal catalytic activity [53, 54]. In addition to pH-responsiveness, Bi_2_O_3_ could offer reactivity in an acidic environment and significantly deplete intracellular glutathione (GSH) intracellularly in TME toward cancer therapy [55, 56]. Despite the success of CT-based contrast agents, Bi-based composites suffer from poor colloidal stability, dose-dependent toxicity and pharmacokinetic shortcomings, leading to poor therapeutic monitoring. To improve the performance of Bi-based constructs, previous studies demonstrated the combination of bismuth with other metal constructs for synergistic tumor theranostics [57, 58], specifically, Cu/Bi-based bimetallic sulfides [59]. In most instances, the versatile nanomaterials were prepared by doping Bi species to influence their weak CT signals, which, in turn, affected the PTT effect of copper chalcogenides. In a case, Lu and colleagues fabricated Cu_1.94_S-Bi_2_S_3_@polymer for combinatorial tumor theranostics, resulting in excellent photothermal conversion and CT imaging abilities [60]. In another instance, Tao and coworkers designed a formulation using biocompatible BSA to *in situ* chelate Cu/Bi with diallyl trisulfide on its surface [61]. In addition to the high X-ray attenuation coefficient of Bi to enhance the radiotherapy approach, thioacetamide (TAA) further transformed Cu/Bi into disulfide nanodots. Nevertheless, the presence of polysulfides could lead to various issues with drug metabolism and biosafety. Although most of the studies fabricated nanometric domains, interestingly, 3D nanoflowers (BiCuOS) were prepared to achieve synergistic CDT/PTT/chemotherapy against cancer [62]. Nevertheless, the inhibitory effect of the materials alone without drugs on cancer cell growth was relatively insignificant. Thus, it is important to explore intelligent nanoarchitectonics without additional therapeutic load and reduced toxicity.

Motivated by these aspects, this study is aimed to demonstrate the fabrication of drugless/drug-like nanoarchitectures based on CuS-nanoplated Bi_2_O_3_ (Bi_2_O_3_@CuS, shortly BC) composites for integrated cancer theranostics (photothermal and chemodynamic therapies, as well as CT imaging) against TNBC. Initially, the spherical-shaped Bi_2_O_3_-based nanoconstructs were prepared using the hydrothermal method. Further, a layer of resin was deposited to fabricate CuS on the surface of Bi_2_O_3_ using the sacrificial template method by oxidizing the surface-coated resin to obtain Bi_2_O_3_@CuS nanocomposites.

**Figure 1.**
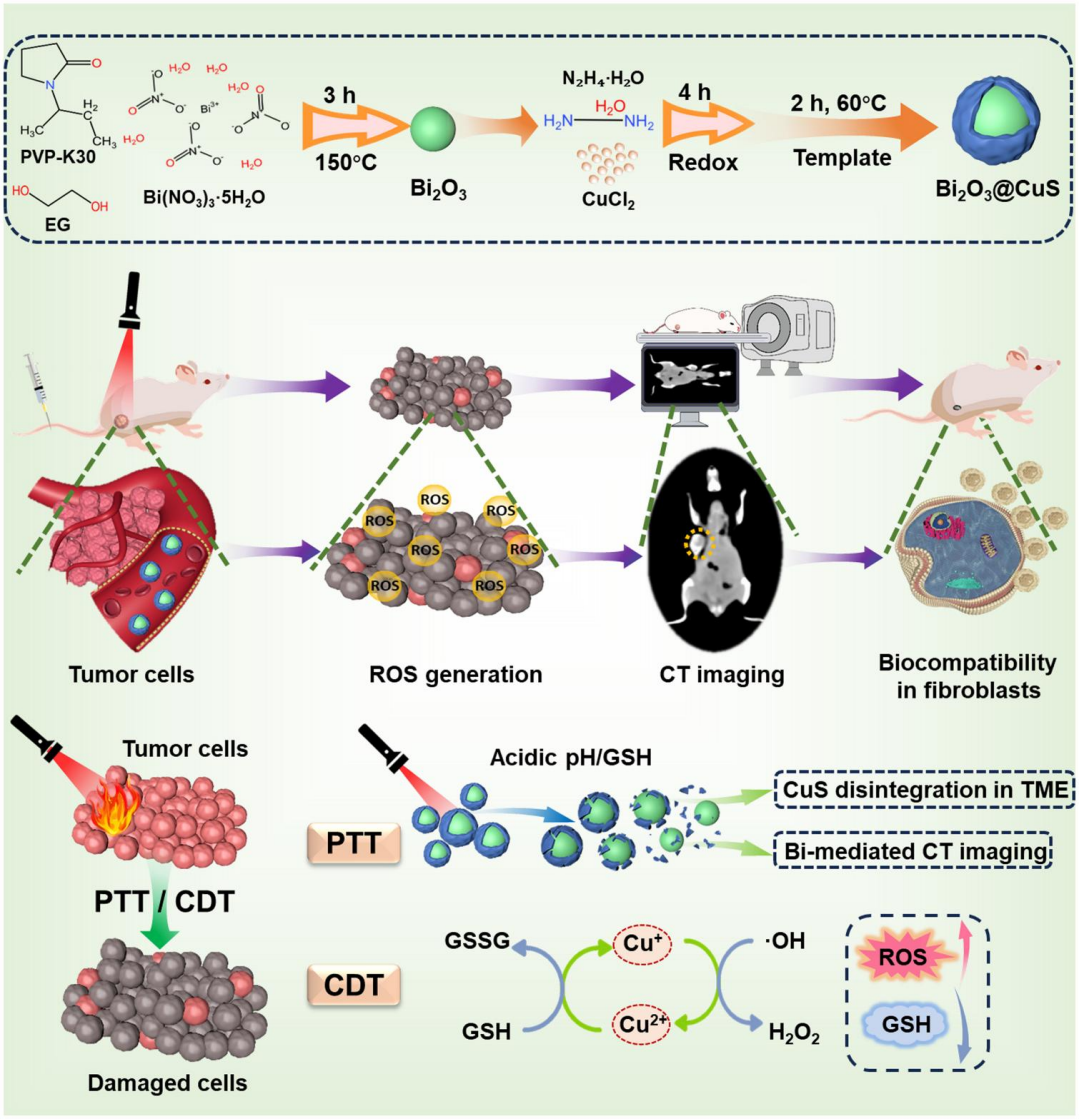
Schematic illustrating the outline of Bi_2_O_3_@CuS composites and their bioefficacy along with molecular mechanisms. A series of steps presenting the synthesis and performance fate (mechanism of action) of Bi_2_O_3_@CuS composites as drugless nanoarchitectures in vivo for integrated tumor photothermal/chemodynamic/CT theranostics against breast carcinoma.

Figure 1 illustrates the *in vivo* synergy of Bi_2_O_3_@CuS nanocomposites in terms of performance and fate in the physiological fluids. On the one hand, the deposited CuS on the Bi_2_O_3_ surface under NIR laser irradiation produced a PTT effect in the TME precisely toward cancer ablation. On the other hand, Bi^3+^ could be monitored in real-time by using a CT imager, which could be helpful in guiding the tumor treatment. Unlike elemental doping, the designed composites with progressive TME-assisted degradation of CuS could reduce the interference in the CT signals of Bi_2_O_3_, demonstrating excellent CT imaging capabilities. In conclusion, the designed Bi_2_O_3_@CuS nanoarchitectonics, with monitoring and guidance of CT imaging, could achieve multimodal therapy (PTT/CDT), providing a novel and effective method for TNBC treatment.

## Experimental section

### Materials

Anhydrous copper sulfide (CuCl_2_·2H_2_O, 99.99%) and bismuth nitrate pentahydrate (Bi(NO_3_)_3_·5H_2_O, 99%) were purchased from McLean Co. Ltd (Hyderabad, India) Polyvinylpyrrolidone-K30 (PVP-K30), anhydrous sodium sulfide (Na_2_S, 95%), 3,3′,5,5′-tetramethylbenzidine (TMB, ≥99%, HPLC), sodium hydroxide (NaOH, 98%) and resorcinol (≥99.5%) were obtained from Aladdin Co. Ltd (Shanghai, China). Hydrazine hydrate (N_2_H_4_, 80%) and concentrated nitric acid (HNO_3_, 65–68%) were purchased from Xilong Science Co., Ltd (Shantou, China). Deionized water was prepared by Millipore Systems (Millipore, MA, USA). Phosphate-buffered saline (PBS) and high glucose-Dulbecco’s modified Eagle medium (DMEM) were purchased from Biological Industries (Kibbutz Breit Hamek, Israel). Streptomycin-penicillin Mix, ethylenediaminetetraacetic acid trypsin and fetal bovine serum (FBS) were purchased from ThermoFisher Scientific Inc. (Waltham, USA). ROS assay and Calcein AM/propidium iodide (AM/PI) kits were purchased from Beyotime Biotechnology Co., Ltd (Shanghai, China). Cell Count Kit (CCK)-8 was purchased from Kaiji Biotechnology Co., Ltd (Taizhou, China). Other related chemicals, solvents and reagents, such as methylene blue Trihydrate (MB), ethylene glycol (EG), hydrochloric acid (36.0–38.0%) and anhydrous ethanol (≥99.7%), were acquired from China National Pharmaceutical Group Chemical Reagent Co., Ltd (Shanghai, China).

### Synthesis of Bi_2_O_3_ nanoconstructs

Bi_2_O_3_ nanospheres were prepared using a hydrothermal method at an optimized temperature by following the reported procedure [63]. Initially, 0.36 g of Bi(NO_3_)_3_·5H_2_O was added to 10 ml of 1 mol·l^−1^ nitric acid solution. After stirring the solution till it becomes colorless and transparent for a while, 0.11 g of NaOH, 50 ml of EG and 1.0 g of PVP-K30 were added subsequently. Further, the solution was mixed thoroughly and transferred to a teflon-lined autoclave for hydrothermal procedure. Then, the autoclave was placed in an oven maintained at a temperature of 150°C for 3 h. After cooling to room temperature, the resultant Bi_2_O_3_ nanospheres were collected and washed 3 times with deionized water.

### Synthesis of Bi_2_O_3_@CuS nanoarchitectures

To fabricate a CuS layer on the surface of Bi_2_O_3_, the redox-assisted sacrificial templating approach was employed. Initially, the prepared Bi_2_O_3_ nanoparticles were dispersed in deionized water, referred to as solution A. Then, resorcinol was dissolved in deionized water labeled as solution B. Further, solution A was slowly added to solution B and stirred thoroughly. Further, the resultant solution was added with formaldehyde (40%) and ammonia (25 wt%) for 12 h at room temperature to obtain a milky white liquid, referred to as solution C. Subsequently, solution C was washed and redispersed in 50 ml of deionized water. PVP-K30 and a certain amount of anhydrous CuCl_2_ were added, followed by a reaction of 5 min. Then, 80% hydrazine hydrate was added, followed by a certain period of oxidation-reduction reaction and the addition of 0.06 g of sodium sulfide at 60°C for 2 h. Finally, the resultant BC nanoconstructs were centrifuged and washed thrice with water and alcohol. To explore the bioavailability of the designed composites, fluorescein isothiocyanate (FITC) was conjugated by magnetically stirring with Bi_2_O_3_@CuS nanoparticles.

### Characterizations

To explore the morphology of the designed composites, transmission electron microscopy (TEM, FEI Talos F200, Thermo Fisher Scientific, Waltham, USA) and scanning electron microscopy (SEM, SU5000, HITACHI Ltd, Tokyo, Japan) instruments were employed. To determine the phase potential (0.1 mg/ml of sample) and fluid dynamics, particle size, surface charge and hydrodynamic state data were collected using phase analysis scattering (PALS) and dynamic light scattering (DLS) techniques using NanoBrook Omni (Brookhaven, NY, USA), respectively. In addition, the particle size distribution was determined by Nano Measurer 1.2 software (Shanghai, China) by randomly selecting 200 nanoparticles. Using an X-ray photoelectron spectrometer (XPS, Escalab 250Xi, Thermo Fisher Scientific, USA), the valence state distribution and actual chemical/elemental composition of each element in the designed Bi_2_O_3_@CuS composites were analyzed. XRD patterns were recorded on SmartLab (Rigaku, Tokyo, Japan) to determine the crystalline patterns of the synthesized composites. TU-1810SPC (Persee, Beijing, China) instrument was used to record UV visible (400–1000 nm) absorbance values.

### Photothermal performance

The photothermal performance of the designed composites was demonstrated by irradiating aqueous dispersions of Bi_2_O_3_ and Bi_2_O_3_@CuS at different concentrations (12.5, 25, 50, 100 and 200 μg·ml^−1^) for 6 min under an NIR laser (808 nm). Subsequently, the role of laser intensity was determined by irradiating aqueous dispersion (200 μl) of Bi_2_O_3_@CuS composites with NIR light at different irradiation powers (0.5, 1, 1.5 and 2 W·cm^−2^). Meanwhile, the temperature and images during the irradiation process were recorded using an infrared thermal imager (Hikvision H16). The photothermal conversion efficiency (η) was calculated. To determine the photothermal stability, illuminated aqueous dispersion of Bi_2_O_3_@CuS (200 μg·ml^−1^) with an 808-nm laser (1 W·cm^−2^) after 6 min was subjected to multiple times by turning off the laser and cooling it naturally to ambient temperature for 4 times, recording the temperature every 30 s.

### *In vitro* CDT effect

The CDT effect of the designed BC composites was determined by measuring the generated •OH radicals *in vitro* using the UV spectrophotometer based on MB and TMB probes, respectively. Briefly, the designed BC composites (200 μg·ml^−1^) under different pH conditions were incubated with MB (10 μg·ml^−1^) and H_2_O_2_ (6 mM) at 37°C in a shaker for 30 min and recorded UV-vis reading. Subsequently, the roles of H_2_O_2_ and GSH at their altered concentrations, i.e. H_2_O_2_ (0, 3, 6, 9 and 12 mM) and GSH (0, 0.5, 1.0 and 2.0 mM) and a constant concentration of aqueous Bi_2_O_3_@CuS solution, were determined under weakly acidic conditions. In addition, the influence of different Bi_2_O_3_@CuS concentrations (0, 25, 50, 100 and 200 μg·ml^−1^) on the degradation of MB in the presence of light was determined. To further validate the type of ROS generation, electron spin resonance (ESR, EMXplus, Bruker, Germany) spectroscopy was used by applying 5,5-dimethyl-1-pyrroline N-oxide (DMPO) as a trapping agent that could capture •OH radicals. In the presence of an acidic H_2_O_2_ solution, a DMPO trap agent was added to Bi_2_O_3_@CuS at a concentration of 200 μg·ml^−1^. Further, the ESR capture signals were recorded after 2 and 5 min. Meanwhile, the validation of singlet oxygen generation (^1^O_2_) was carried out using a UV-vis spectrometer utilizing DPBF to treat aqueous Bi_2_O_3_@CuS solution (200 μg·ml^−1^). The changes in the absorbance values of the solution at 350–550 nm range were recorded within 0–5 min after light irradiation (1 W·cm^−2^).

### GSH depletion

The 5,5′-dithiobis-(2-nitrobenzoic acid) (DTNB) indicator was used to evaluate the intracellular depletion ability of GSH by Bi^3+^ ions. After adding GSH to the four experimental groups, namely Bi_2_O_3_, CuS, Bi_2_O_3_@CuS and PBS without nanoparticles (Control, pH −5.5) for 12 h, the supernatant was centrifuged and added to 96-well plates (*n* > 3). After incubating with 30 μl of DTNB solution in a shaker for 5 min, the absorbance values of each well at 412 nm were measured using the microplate reader.

### *In vitro* degradation

Initially, the designed Bi_2_O_3_@CuS constructs were added to PBS buffer (pH-5.5 PBS+2 mM GSH (5 μl)+4 mM H_2_O_2_ (5 μl), a total of 1 ml) that simulated the *in vivo* TME and incubated in a shaker. Further, the changes in the solution color were photographed and compared at different reaction durations (0, 3, 6, 12 and 24 h) to observe the degradation of Bi_2_O_3_@CuS from a macroscopic point-of-view.

### *In vitro* anticancer studies

#### Cell line

Breast cancer cells (4T1 cell line) and normal fibroblasts (L929 cell line) from mice (Wuhan Punosai Life Technology Co., Ltd, Wuhan, China) were incubated in high glucose DMEM containing 10% FBS and 10% penicillin/streptomycin in a humidified 5% CO_2_ atmosphere incubator maintained at 37°C.

#### Cytotoxicity assay

CCK-8 assay was employed to evaluate the cytotoxicity rate of the designed nanoparticles quantitatively. Briefly, 4T1 and L929 cell lines were seeded into 96-well plates at a cell density of 8 × 10^3^ per well, respectively, and incubated overnight for proper attachment. 4T1 cells were subjected to different treatments and divided into non-laser and laser groups. Further, the cells were added with corresponding sample groups (Bi_2_O_3_ and Bi_2_O_3_@CuS) at various concentrations (0, 12.5, 25, 50, 100 and 200 μg·ml^−1^). The laser treatment group of cells was irradiated with 808 nm laser (1 W·cm^−2^) for 6 min after 4 h of sample administration and incubated. The CCK-8 working solution (1:10 (v/v) in fresh complete medium) was added under dark conditions. After incubation for 60–90 min, the absorbance values at a wavelength of 450 nm were detected using a microplate reader (1510, Thermo Fisher Scientific), and the cell survival rate was calculated as stated below.

$$\text{Cell}\;\text{viability}\;\left(\%\right)=\left({\text{Abs}}_{\text{Treatment}}/{\text{Abs}}_{\text{Control}}\right)\times 100$$

#### Live and dead staining

Briefly, 4T1 cells were seeded (1 × 10^5^ cells per well) into 24-well plates and incubated at 37°C for 24 h. Further, the cells were incubated with Bi_2_O_3_ and Bi_2_O_3_@CuS samples (200 μg·ml^−1^) for 12 h. Accordingly, the laser treatment group was irradiated with a laser for 6 min. After incubation for 8 h, the cells were washed with PBS twice and added with Calcein AM/PI in the culture medium prepared at a ratio of 1:1:1000 for 30–60 min in the dark environment. Finally, the cell fluorescence images were captured by an inverted fluorescence microscope (Axio observer A1, Zeiss, Oberkochen, Germany).

#### ROS generation assay

4T1 cells were seeded at a density of 1 × 10^5^ cells per well into a 24-well plate and cultured under the cell incubator for 24 h. Further, 4T1 cells were incubated with the samples of Bi_2_O_3_ and Bi_2_O_3_@CuS at a concentration of 200 μg·ml^−1^, and corresponding wells were treated with 808 nm laser for 6 min and incubated for 12 h. After giving a PBS wash, the 2,7-dichlorodihydrofluorescein diacetate (DCFH-DA) working solution prepared in medium (10 mM, 200 μl) was added to the cells and incubated for 20 min in the dark. Finally, the fluorescence images (Ex/Em—488/525 nm) were captured using an inverted fluorescence microscope.

#### Intracellular GSH depletion

4T1 cells adhered to a 6-well plate were incubated with various samples of DMEM, PBS, Bi_2_O_3_ (200 μg·ml^−1^) and Bi_2_O_3_@CuS (200 μg·ml^−1^) for 8 h, respectively. Subsequently, cells from different treatment groups were collected in tubes using a scraper and subjected to cell fragmentation treatment. Further, the intracellular GSH content was measured using a reduced GSH assay kit.

#### Mitochondrial membrane potential

4T1 cells were seeded in 12-well plates and allowed to adhere to the bottom of the plates by incubating them for 24 h. After incubating with various samples of Bi_2_O_3_ and Bi_2_O_3_@CuS at 200 μg·ml^−1^, JC-1 staining solution was added to the cells for 8 h and then subjected to PBS washes 3 times. Finally, cells were captured using CLSM (TCS Sp8, Leica, Germany).

### *In vivo* investigations

#### Animal grouping and treatment

The experimental animals, i.e. healthy BALB/c female mice, aged 6–8 weeks and weighed about 20 g, were purchased from Wushi Laboratory Animals Co., Ltd, and maintained in well-ventilated cages by providing sterile water and pellet food *ad libitum*. All animal-related experiments were carried out in strict accordance with the guiding principles of the Institutional Animal Care and Use Committee of Huaqiao University (Approval No. A2020029) and following the guidelines from the Administration of Affairs Concerning the Experimental Animals of China. Initially, 4T1 cancer cells (100 μl) were inoculated subcutaneously to establish the tumor model. After attaining the tumor volume (V) of ∼100 mm^3^ (measured as total width (W) and total length (L), according to the formula *V*=(*W*^2^×*L*)), the mice were randomly divided into five groups with five mice in each group (*n* = 5), including (1) Control group (normal saline), (2) Light group (normal saline), (3) Bi_2_O_3_, (4) Bi_2_O_3_@CuS and (5) Bi_2_O_3_@CuS+Laser. Mice in different treatment groups were intravenously injected with corresponding samples at the injection dosage controlled at 10 mg·kg^−1^. In the light irradiation treatment group, the tumor site in mice was irradiated with an 808-nm laser for 6 min after 12 h of injection of the sample. Further, the temperature changes and infrared images of the tumor area under different irradiation times were recorded with an infrared thermal imager. At the predetermined time intervals, the body weight and tumor volume of each group of mice were recorded every 2 days, and intravenous administration was carried out every 2 days to evaluate the efficacy of the designed composites.

#### Hemolysis assay

Fresh blood samples (1.0 ml) drawn from normal mouse eyeballs were mixed with PBS (9.0 ml) and gently shaken for uniform mixing. After centrifugation (1500 rpm, 5 min), the supernatant serum was discarded, and the obtained red blood cells (RBCs) were washed thrice with PBS. Further, a tube of pure RBCs (solution A, 2 ml) and a tube of 8 ml of 10% RBCs in PBS (solution B). Typically, the samples of the positive group (total hemolysis A), the negative group (solution B vs normal saline at a volume ratio of 1:1) and the experiment group (Bi_2_O_3_@CuS at different concentrations) were incubated for 2 h at 37°C. After uniform mixing, each tube was centrifuged (2500 rpm, 5 min), and the supernatant (100 μl) was carefully collected into a 96-well plate. The absorbance values at 540 nm were determined by scanning the plate using a microplate reader, and the hemolysis rate was calculated as follows:

$$\begin{array}{c} \text{Hemolysis}\;\text{assay}\;(\%)\\ =\frac{\text{Absorbance}\;\text{of}\;\text{sample}-\text{negative}\;\text{absorbance}}{\text{Positive}\;\text{absorbance}-\text{negative}\;\text{absorbance}}\times 100 \end{array}$$

#### Histochemical staining and biochemical analyses

In addition to the hemolysis assay, histological changes were elucidated using histochemical staining and several other biochemical analyses. Briefly, the mice of all the treatment groups were sacrificed after 15 days of treatment. Further, the weight and volume of the excised tumors were recorded and photographed. The tumors and major organs (heart, liver, lung, kidney and spleen) of mice from different treatment groups were collected for histopathological analysis by hematoxylin and eosin (H&E) staining. The stained tissue sections were observed under a microscope to analyze their histological changes. To further verify the biological safety and therapeutic effect of nanomaterials, blood from the eye of three randomly selected mice in each group was collected to analyze blood routine, liver function, kidney function and other indicators.

#### CT imaging

The CT imaging performance of Bi_2_O_3_@CuS nanocomposites was evaluated using a CT scanner (Bruker micro-CT, SkyScan1276). Briefly, different concentrations of Bi_2_O_3_ and Bi_2_O_3_@CuS composites (0, 0.63, 1.25, 2.5, 5.0, 10 and 20 μg·ml^−1^) in the PBS solution at different pH values (pH = 5.5 and 7.4) were prepared. Prior to *in vivo* CT performance, *in vitro* X-ray CT imaging of Bi was determined. Then, Bi_2_O_3_@CuS solution (5 mg·ml^−1^, 100 μl) was administered intravenously in the tumor-bearing mice for *in vivo* CT imaging. Further, the CT images before and after injection (3 and 6 h) were recorded, respectively. The imaging parameters were set as follows: voltage of 80 kV, current of 100 μA, single exposure time of 50 ms, scanning resolution of 25 μm and scanning angle interval of 0.5°.

#### Drug metabolism of different nanoarchitectures

To demonstrate the drug metabolism ability of Bi_2_O_3_ and Bi_2_O_3_@CuS *in vivo*, urine samples were collected from each group of tumor-bearing mice at different time intervals (0, 12 and 24 h) after injection of saline, Bi_2_O_3_ and Bi_2_O_3_@CuS, respectively. The urine samples from each treatment group of animals at different time intervals were then sequentially added with 10 μl of MB solution and 2 mM GSH and incubated in a shaker at 37°C for 30 min. The solutions were further arranged and photographed to assess the color change of the solutions.

### Statistical analysis

The experimental data were expressed as mean±SD (*x* ± *s*). The data between multiple groups were compared using one-way analysis of variance (ANOVA), followed by Tukey’s *post hoc* test, considering *P* < 0.05 as statistically significant.

## Results and discussion

### Construction and characterization

**Figure 2.**
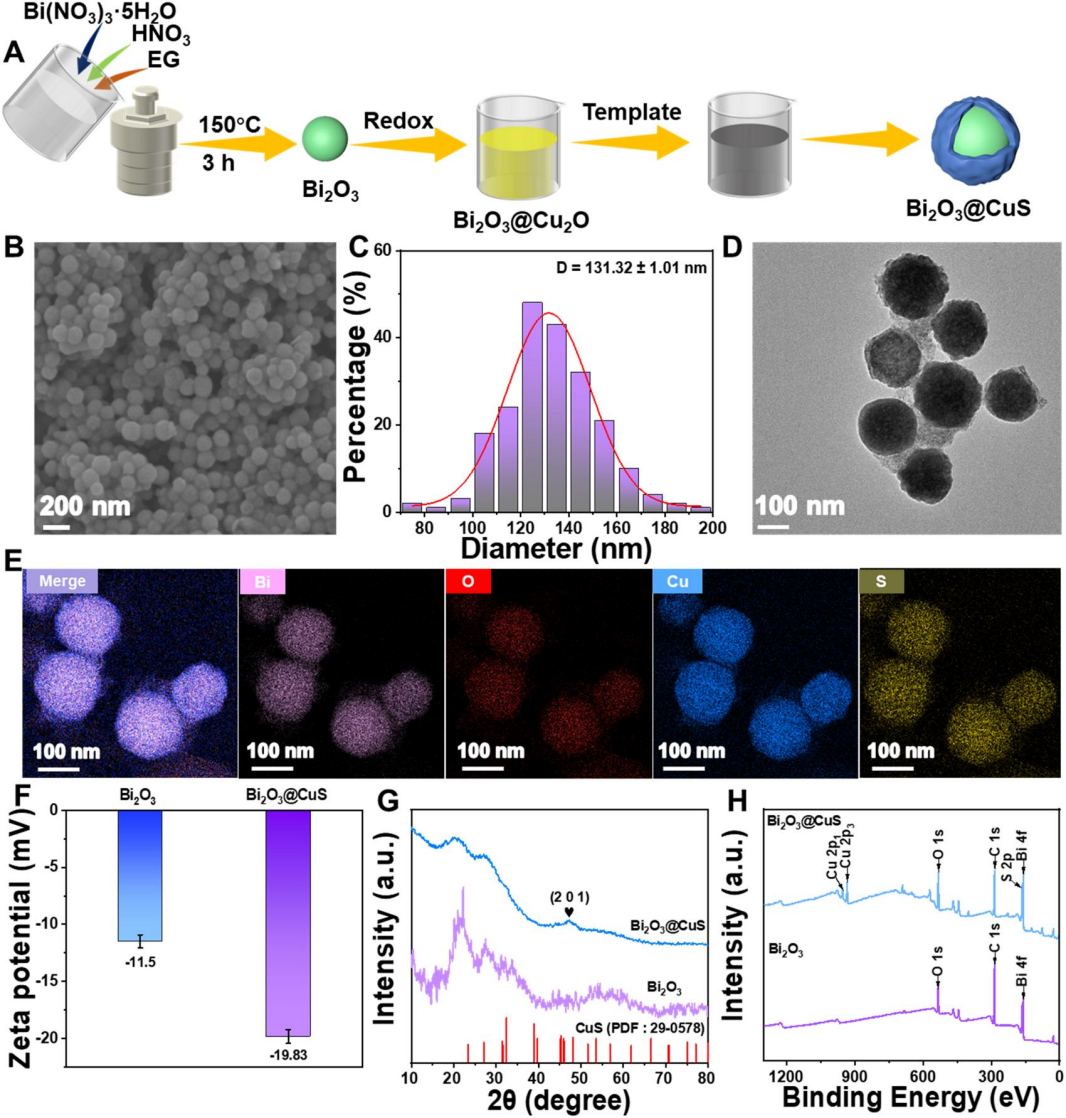
Morphological and physicochemical characterizations of Bi_2_O_3_ and Bi_2_O_3_@CuS nanoarchitectures. (**A**) Schematic diagram illustrating the fabrication of Bi_2_O_3_@CuS nanoarchitectures. (**B**) SEM, (**C**) particle size distribution and (**D**) TEM image of Bi_2_O_3_@CuS nanoarchitectures. (**E**) EDS-based elemental mapping of Bi_2_O_3_@CuS nanoarchitectures. (**F**) Zeta potential values, (**G**) XRD patterns and (**H**) XPS full spectrum of Bi_2_O_3_ and Bi_2_O_3_@CuS nanoarchitectures.

Figure 2A presents a series of detailed steps for the synthesis of Bi_2_O_3_@CuS nanoparticles. Briefly, Bi_2_O_3_ nanospheres were initially synthesized using the hydrothermal approach at an appropriate high temperature. The SEM (Figure S1A) and TEM (Figure S1B) observations showed a spherical shape of formed Bi_2_O_3_ nanospheres with excellent dispersion and relatively monodispersed particles with uniform particle size distribution. Further, the particle size distribution of Bi_2_O_3_ nanospheres was determined by analyzing the nanoparticles (*n* = 200) from the SEM image, resulting in an average size of about 90 nm (Figure S1C).

Prior to CuS nanoplating, optimization experiments were performed in terms of the reaction time of Cu_2_O nanoparticles (2, 4 and 12 h) and the feed ratio of Bi_2_O_3_/CuS (2:1, 1:2 and 1:1) toward synthesizing Bi_2_O_3_@CuS nanoarchitectures [64, 65]. Accordingly, these two influencing experimental conditions as optimization attributes could play crucial roles in the synthesis process, affecting the eventual morphological characteristics. The changes in both conditions would affect the structure and properties of the synthesized nanomaterials, leading to the deprived bioefficacy performance. Firstly, the feeding ratio of Bi_2_O_3_/CuS was tentatively set to 1:1 to validate the reaction time. At the reaction times of 2 and 12 h, an incomplete reaction could be observed, resulting in the transparent coating of the CuS layer and nanoclusters with poor dispersion, respectively (Figure S2A and C). Contrarily, the intermediate reaction time of 4 h resulted in the successful CuS coating over Bi_2_O_3_ nanospheres with uniform particle size and dispersion (Figures S2B and S3C). After confirming the optimal reaction time, the feeding ratio was further determined by altering the amount of one of the precursors. The feed ratios of Bi_2_O_3_/CuS of 2:1 and 1:2 resulted in two different types of nanostructures (Figure S3A and C), including fully formed CuS and etched inner cavities of Bi_2_O_3,_ resulting in hollow CuS structures, respectively. These changes in the morphological attributes could be due to the Kirkpatrick effect between ions. Accordingly, the optimal experimental conditions, i.e. the reaction time of 4 h and the feeding ratio of 1:1 were subsequently applied for synthesizing Bi_2_O_3_@CuS nanoparticles and subjected to physicochemical characterization. As depicted in SEM and TEM observations, the designed Bi_2_O_3_@CuS nanoarchitectures at the optimal conditions retained a spherical shape even after CuS nanoplating (Figure 2B and D). However, the particle size distribution of Bi_2_O_3_@CuS nanoarchitectures indicated a slight increase in the average size to 131 nm (Figure 2C). Further, the TEM-based energy-dispersive spectroscopic (EDS) analysis showed the distribution of Cu, Bi, S and O elements, indicating the successful CuS nanoplating over Bi_2_O_3_ (Figure S1D and Figure 2E). Together, these findings successfully validated the synthesis of Bi_2_O_3_@CuS nanoarchitectures, highlighting the uniform size distribution and elemental analysis.

Further, the DLS measurements presented the changes in the hydrodynamic sizes of Bi_2_O_3_@CuS nanoarchitectures, indicating an increase in the average particle size of Bi_2_O_3_ nanoconstructs from 177 to 207 nm after CuS nanoplating over them (Figure S4A and B). The Zeta potential values by the PALS measurements indicated a change in the surface charge of Bi_2_O_3_ nanoconstructs from −11.5 to −19.83 mV after CuS nanoplating over Bi_2_O_3_ nanoarchitectures (Figure 2F). However, it should be noted that the altered zeta potential value remained within the range of relative colloidal stability of nanoformulations in the physiological fluids, which could be highly conducive for biomedical applications. The XRD patterns and their peak values of Bi_2_O_3_ are consistent with the previous literature [66]. The peak at 46.11° in the Bi_2_O_3_@CuS nanoarchitectures could correspond to the (2 0 1) plane of CuS (PDF: 29-0578) [67], confirming the synthesis of Bi_2_O_3_@CuS (Figure 2G). Further, the atomic state and its composition in the nanoparticles containing Cu, S, Bi and O elements were determined using the XPS analysis. The XPS spectral binding energy was calibrated using the carbon 1 s (C-1s) peak located at 284.6 eV (Figure 2H). From the high-resolution XPS spectrum of Bi (Figure S4C), the spectral peak at 164.6 eV/159.3 eV could be ascribed to Bi 4f_5/2_, indicating the presence of Bi^3+^ in Bi_2_O_3_@CuS. The peak at 162.8 eV could be attributed to Bi, indicating a reduction in Bi ions [68]. In Figure S4D, the peaks at 933.8 and 952.6 eV could correspond to Cu 2p_3/2_ and Cu 2p_1/2_, respectively [69, 70]. Moreover, the O 1s fine spectrum presented that the Bi-O bond was slightly shifted at around 531.3 eV (Figure S4E), which could be due to the interactions between Bi_2_O_3_ and CuS [71]. Considering the valence state of Cu, i.e. Cu^+^ and Cu^2+^, these results indicated that Bi_2_O_3_@CuS nanoarchitectures could offer substantial CDT.

### Photothermal performance

**Figure 3.**
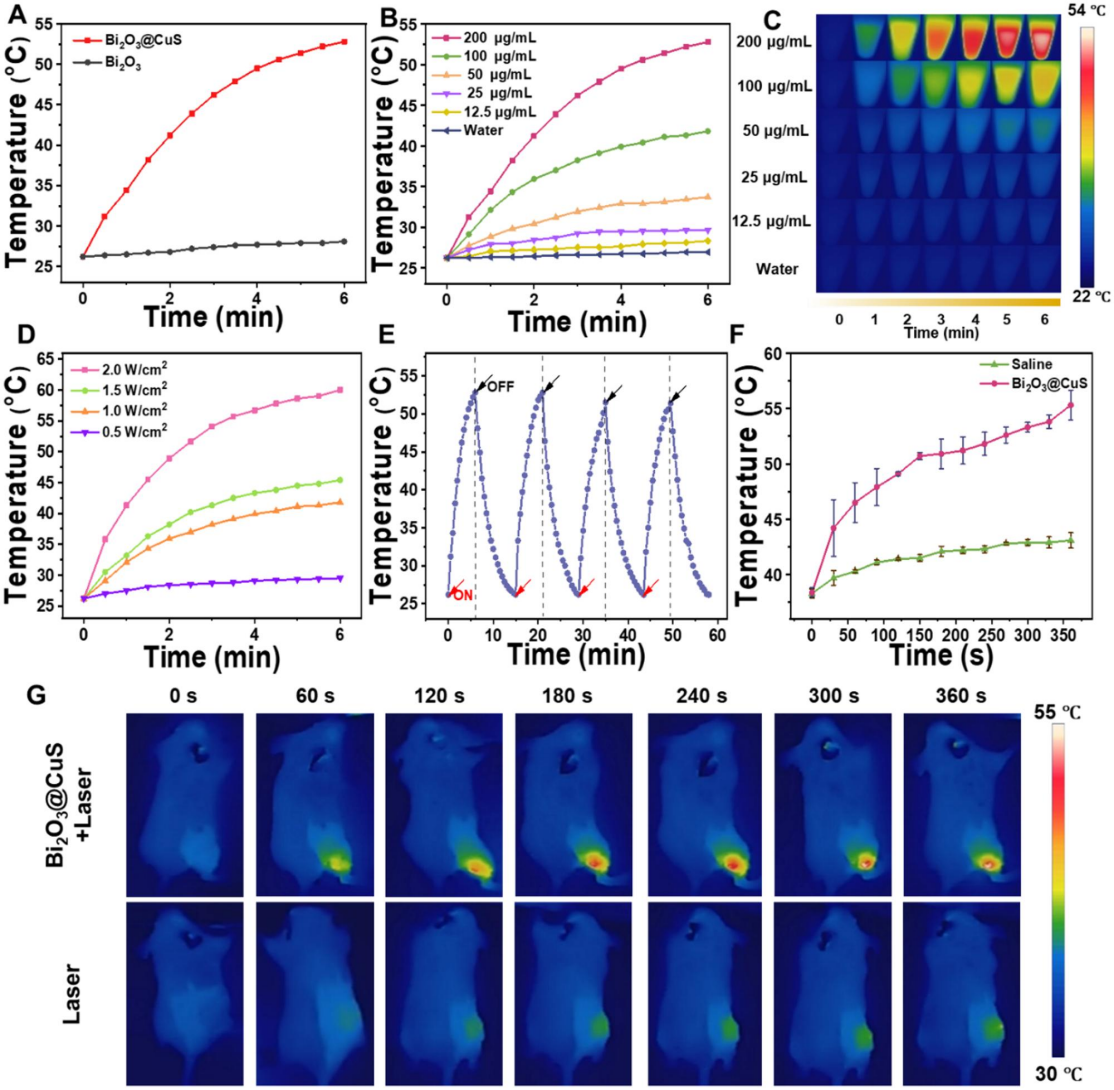
Photothermal properties of Bi_2_O_3_@CuS nanoarchitectures. (**A**) Photothermal temperature rise curves of Bi_2_O_3_ and Bi_2_O_3_@CuS under 808 nm laser irradiation. (**B**) Temperature rise curve of water and different concentrations of Bi_2_O_3_@CuS under 808 nm laser irradiation. (**C**) Images of Bi_2_O_3_@CuS samples at various concentrations show the photothermal temperature rise with time. (**D**) Temperature rise curves of Bi_2_O_3_@CuS (100 μg·ml^−1^) under different power densities of 808 nm laser irradiation. (**E**) The aqueous solution of Bi_2_O_3_@CuS was irradiated by an 808-nm laser for four cycles (1 W·cm^−2^). (**F**) Temperature change curve and (**G**) photothermal imaging map of the tumor-bearing mice tumor site under 808 nm laser (1 W·cm^−2^) irradiation.

Prior to detecting temperature changes *in vitro*, the UV-vis spectroscopy absorbance values of Bi_2_O_3_@CuS in the wavelength range of 400–1000 nm were observed, which indicated significantly higher than that of Bi_2_O_3_ and CuS separately (Figure S4F). Notably, the improved NIR absorption of Bi_2_O_3_@CuS at 808 nm could be due to the sulfide species. Meanwhile, the UV-absorption values of Bi_2_O_3_@CuS at different gradient concentrations (0–200 μg·ml^−1^) attained a linear fitting at 808 nm. Further, the extinction coefficient (α) of Bi_2_O_3_@CuS at 808 nm was calculated according to Lambert’s law to be 1.58 l·g^−1 ^cm^−1^ (Figure S5A). Accordingly, an 808-nm laser (1 W·cm^−2^) was used to analyze the photothermal performance of Bi_2_O_3_ and Bi_2_O_3_@CuS (200 μg·ml^−1^) aqueous solution. After 6 min of irradiation, the temperature of the Bi_2_O_3_@CuS sample solution (55°C) was higher than the Bi_2_O_3_ constructs, indicating excellent photothermal properties of the CuS-coated Bi_2_O_3_ constructs (Figure 3A).

Further, the PTT effect of aqueous Bi_2_O_3_@CuS sample was determined by exposing different concentrations (0–200 μg·ml^−1^). As depicted in Figure 3B, a rise in the temperature was observed with an increase in the concentration of Bi_2_O_3_@CuS sample, in which the temperature increased by 30°C at 200 μg·ml^−1^ after irradiation for 6 min. In addition, the infrared images of the temperature at different concentrations and different time intervals were captured with an infrared tester (Figure 3C). Moreover, the thermal image recordings showed a proportional rise in the temperature of Bi_2_O_3_@CuS sample with an increase in the power density (0–2 W·cm^−2^, Figure 3D). To determine the photothermal stability, the laser was turned off after irradiating (808 nm, 1 W·cm^−2^) Bi_2_O_3_@CuS aqueous solution for 6 min, and the temperature of natural cooling was recorded every 30 s and repeated 4 times. As depicted in Figure 3E, the Bi_2_O_3_@CuS nanocomposites exhibited tremendous photothermal stability with no substantial influence on the temperature. Nevertheless, a slight reduction in the temperature after two cycles was observed, which could be due to the decomposition of the outer CuS layer under continuous photothermal irradiation. Simultaneously, the photothermal conversion efficiency was calculated to be 37.09% (Figure S5B). Notably, the calculated photothermal conversion efficiency was higher than that of other photosensitizers for PTT, such as black phosphorus quantum dots (28.4%) [72] and Au@Bi_2_Se_3_ (35.5%) [73]. Meanwhile, the PTT efficacy *in vivo* also enabled photothermal imaging (PTI) for tumor diagnosis. Accordingly, the photothermal observations were recorded in tumor-bearing mice in the control and treatment groups every 30 s within 6 min, indicating a consistent photothermal trend with that of *in vitro* findings (Figure 3F). The infrared images of the animals administered with the designed composites were captured after different periods and compared with the control group (Figure 3G). The temperature at the tumor site in the Bi_2_O_3_@CuS sample treatment group increased by 25°C. In contrast, the control group showed only a rise of 4.6°C, indicating the potential of Bi_2_O_3_@CuS as an anti-tumor PTT formulation.

### CDT efficacy

**Figure 4.**
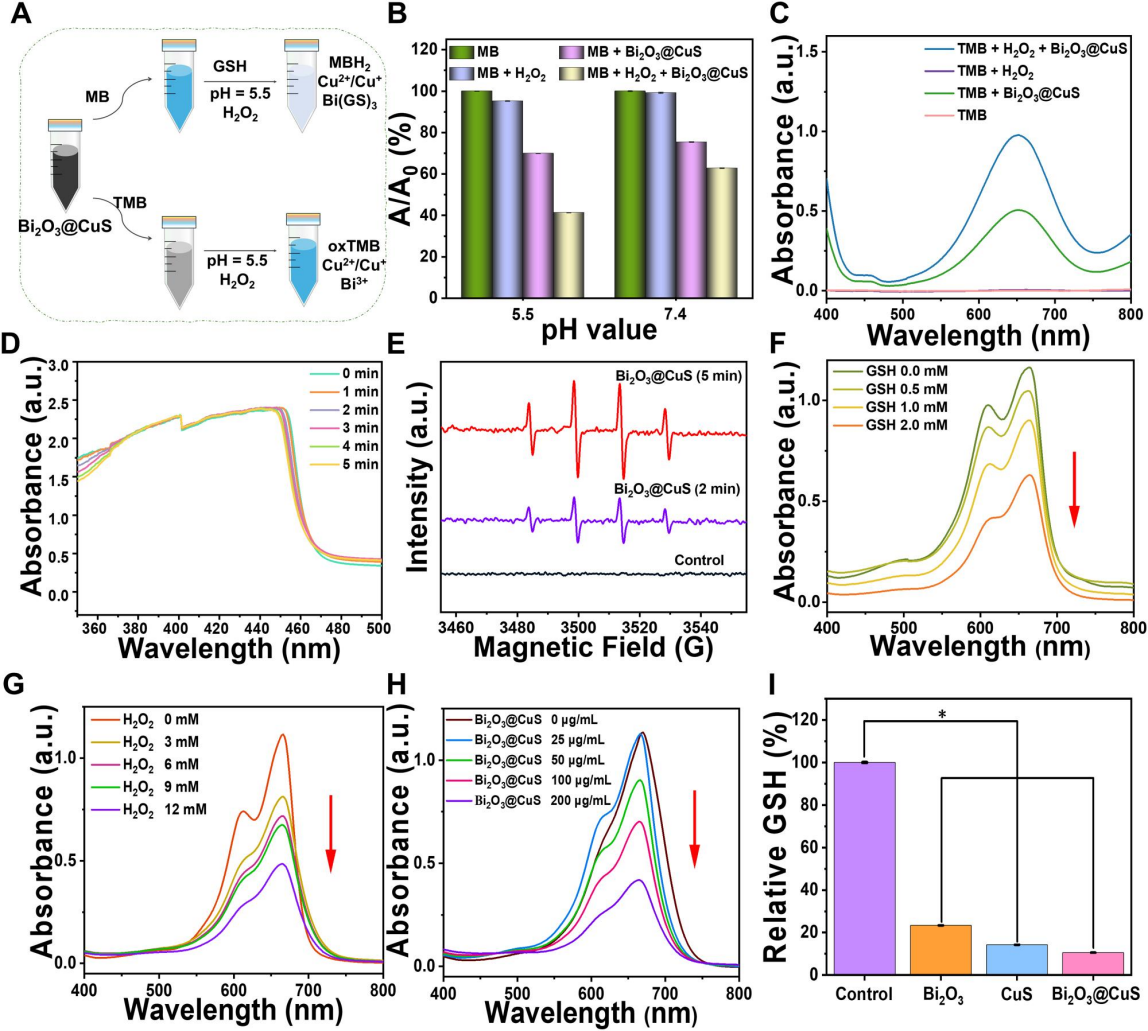
CDT Efficacy of Bi_2_O_3_@CuS nanoarchitectures in vitro. (**A**) Schematic illustrating the CDT-assisted free radical generation process of Bi_2_O_3_@CuS nanoarchitectures. (**B**) The absorption rate of degraded MB at 665 nm under various pH values at different time intervals. (**C**) The oxidation of TMB corresponds to the (**B**) diagram at pH—5.5. (**D**) The image shows the singlet oxygen (^1^O_2_) generation efficacy by Bi_2_O_3_@CuS nanoarchitectures. (**E**) ESR spectra of •OH captured by DMPO of Bi_2_O_3_@CuS. MB degradation under different concentrations (**F**) GSH, (**G**) H_2_O_2_ and (**H**) Bi_2_O_3_@CuS. (**I**) The GSH depletion efficacy by different nanoparticles in vitro using DTNB. * represents *P* < 0.05.

Typically, copper-based biomaterials evidently demonstrate that copper ions could mediate the Fenton-like reaction, producing highly toxic ROS intracellularly in tumors, inducing the CDT effect in the TME. In this study, colorimetric agents, such as MB and TMB probes, were used to verify the Fenton-like reactivity of Bi_2_O_3_@CuS, which could induce CDT to produce toxic ROS in tumors. MB in aqueous solution reduces to dihydromethylene blue (MBH_2_), changing the solution color from blue to colorless, with a characteristic peak at 665 nm. TMB can be catalyzed to produce blue oxTMB with an absorption peak at 652 nm under mildly acidic conditions (Figure 4A). As shown in Figure 4B and C, the corresponding absorption peaks of MB and TMB showed their degradation in the presence of H_2_O_2_, indicating that Bi_2_O_3_@CuS could catalyze H_2_O_2_ to generate ROS in the mild acidic environment. The characteristic peak absorption value at pH—5.5 decreased to less than 40% of the control group after adding MB in the reaction system containing designed nanoconjugates and H_2_O_2_. In contrast, the reduction of characteristic peak absorption value in the neutral environment (pH—7.4) after adding MB was lesser than in the at pH—5.5 microenvironment. Moreover, TMB probes exhibited similar results, indicating that Bi_2_O_3_@CuS could provide excellent catalytic efficiency in the acidic microenvironment, generating large amounts of ROS.

Further, the type of ROS generated by Bi_2_O_3_@CuS nanoarchitectures was determined. Initially, an investigation was conducted on the singlet oxygen (^1^O_2_) species by setting up different samples, including negative (pure water), positive (IR780), Bi_2_O_3_ and Bi_2_O_3_@CuS treatment groups. From Figure S6B, it was observed that the absorbance value of the positive treatment group gradually decreased at 400–440 nm with an increase in the laser irradiation time, indicating the generation of singlet oxygen under illumination. Nevertheless, the experimental treatment (Bi_2_O_3_ and Bi_2_O_3_@CuS) groups showed no absorption at 400–440 nm (Figure 4D and Figure S6C), which was similar to the trend of the negative control group (Figure S6A), indicating that these composites could not produce ^1^O_2_ under acidic conditions and light exposure. In addition, ESR spectroscopy was employed to investigate the ROS generated by Bi_2_O_3_@CuS using DMPO as •OH radical scavenger. The signal peak captured by DMPO was 1:2:2:1 (Figure 4E), increasing the captured intensity with time, indicating the production of •OH radicals.

In addition to mildly acidic pH, the intracellular GSH concentration is relatively high in the tumor environment, which may influence the therapeutic efficacy of the designed nanoconjugates. The degradation ability of MB was increased with a rise in the GSH concentration, showing an overall downward trend (Figure 4F). Similarly, the resultant influence of other components was explored by exposing GSH to different concentrations of H_2_O_2_ and Bi_2_O_3_@CuS. The absorption value of MB at about 665 nm decreased significantly, indicating that Bi_2_O_3_@CuS could produce •OH radicals in the acidic environment containing H_2_O_2_ and GSH (Figure 4G and H). In addition to CuS reacting with GSH due to Fenton-like reactivity, Bi^3+^ would deplete GSH in an acidic environment [74]. Herein, DTNB reagent was used to carry out GSH depletion experiments using different nanoparticles. Accordingly, the GSH levels were relatively lower in the Bi_2_O_3_ treatment group, as Bi^3+^ species could react with GSH in an acidic environment (Figure 4I) [75]. Further, Bi_2_O_3_@CuS showed a lower concentration of GSH in the solution than Bi_2_O_3_ and CuS samples. Accordingly, the ROS removal would be greatly reduced with the GSH depletion, resulting in increased ROS. These findings indicated that Bi_2_O_3_@CuS could induce synergistic CDT effect and deplete GSH in TME toward generating ROS for improved cancer therapy.

After successfully exploring the stimuli (pH/GSH)-responsiveness, the colloidal stability in terms of dispersion and suspension abilities of the designed Bi_2_O_3_@CuS composites was demonstrated. Notably, this physicochemical attribute plays a crucial role in biosafety and degradability due to the need for applicability *in vitro* and *in vivo*. The colloidal stability of the nanoparticles at different concentrations was determined by dispersing them in aqueous solutions and serum (FBS)-containing DMEM, respectively. As shown in Figure S7A, the color of the solution containing Bi_2_O_3_@CuS composites darkened gradually with concentration. Moreover, the Bi_2_O_3_@CuS at the concentrations of 200 and 400 μg/ml were well dispersed in aqueous and serum (FBS)-containing DMEM with no signs of aggregation, indicating their colloidal stability. As notified earlier, the CuS nanoplating over the surface of Bi_2_O_3_ could improve their theranostic efficacy as a CT imaging contrast agent both *in vitro* and *in vivo*. Therefore, the degradation behavior of the designed Bi_2_O_3_@CuS composites was validated by incubating them in the simulated TME environment *in vitro* and referring to the change in color. After 24 h of reaction time, the solution color showed a clear light gray solution, indicating the pH/GSH-responsive behaviors of the coated CuS (Figure S7B). The degradable CuS could substantially expose Bi_2_O_3_ constructs and further transform Bi_2_O_3_ to Bi^3+^ in the acidic environment, thus exhibiting excellent CT imaging toward real-time imaging and subsequent therapeutic effects.

### *In vitro* investigations

**Figure 5.**
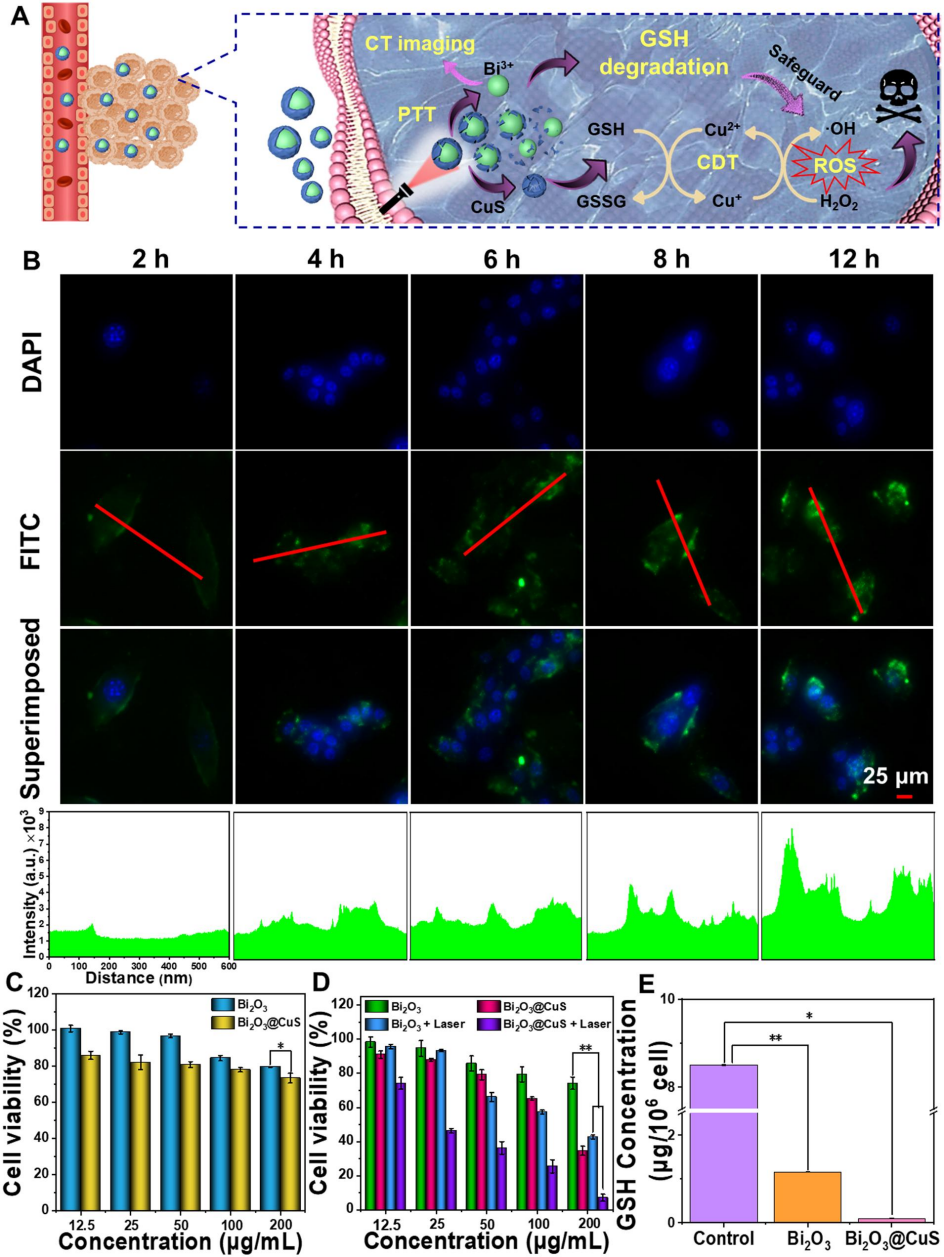
In vitro cell uptake study and tumor ablation effects of Bi_2_O_3_@CuS composites. (**A**) Schematic diagram illustrating intracellular mechanism toward synergistic CDT-PTT effects. (**B**) CLSM images of FITC-Bi_2_O_3_@CuS co-cultured with 4T1 for 2, 4, 6, 8 and 12 h, and their corresponding quantified levels in the bottom row (the nuclei were counter-stained using DAPI. The laser was irradiated (808 nm) in each corresponding treatment group, time: 6 min, power: 1 W·cm^−2^). CCK-8 assay presenting viabilities of (**C**) L929 cells and (**D**) 4T1 cells under different incubation and treatment conditions. (**E**) Depletion of GSH by different nanoparticles in cells. * represents *P* < 0.05 and ** indicates *P* < 0.01.

The designed Bi_2_O_3_@CuS nanoarchitectonics could react with TME, triggering a series of responsive behaviors toward executing the anti-tumor therapy. We hypothesized that the responsive behaviors of the designed nanocomposites could lead to antitumor potential in 4T1 cells (Figure 5A). Typically, the acid-responsive CuS layer of Bi_2_O_3_@CuS could be decomposed by light and acidic environment, increasing localized temperature and generating dreadful •OH radicals to produce PTT and CDT effects. Further, the exposed Bi_2_O_3_ could be delivered at the tumor site, subsequently depleting GSH intracellularly. Thus, the available GSH levels could be insufficient to oxidize generated •OH radicals, which could act on the damage of intracellular organelles toward executing apoptosis and subsequent therapeutic effect. In addition, the intratumor aggregation of Bi^3+^ ions could facilitate strong CT imaging contrast efficacy, resulting in intense real-time CT imaging. These Bi_2_O_3_@CuS architectures could result in a decrease in the GSH concentration and a substantial increase in ROS production, facilitating improved theranostics.

#### Cellular uptake

The cellular uptake of the designed FITC-conjugated Bi_2_O_3_@CuS composites was demonstrated by co-culturing them with 4T1 cells for different incubation periods (2, 4, 6, 8 and 12 h). As shown in Figure 5B, the intensity of the green fluorescence presenting FITC-Bi_2_O_3_@CuS was enhanced in the cytoplasm after incubating for 4, 6, 8 and 12 h, in a time-dependent manner of the culture with a slight green fluorescence in the nuclei. Specifically, the fluorescence intensity significantly increased after 12 h of incubation, indicating that Bi_2_O_3_@CuS nanoparticles were substantially internalized. Consistently, the quantified green-fluorescence intensity of nanoconjugates showed increased levels over time. Together, it suggested that Bi_2_O_3_@CuS presented an excellent internalization efficacy, providing the possibility of releasing active ions and making them functional in the subsequent TME.

#### Cytotoxicity

**Figure 6.**
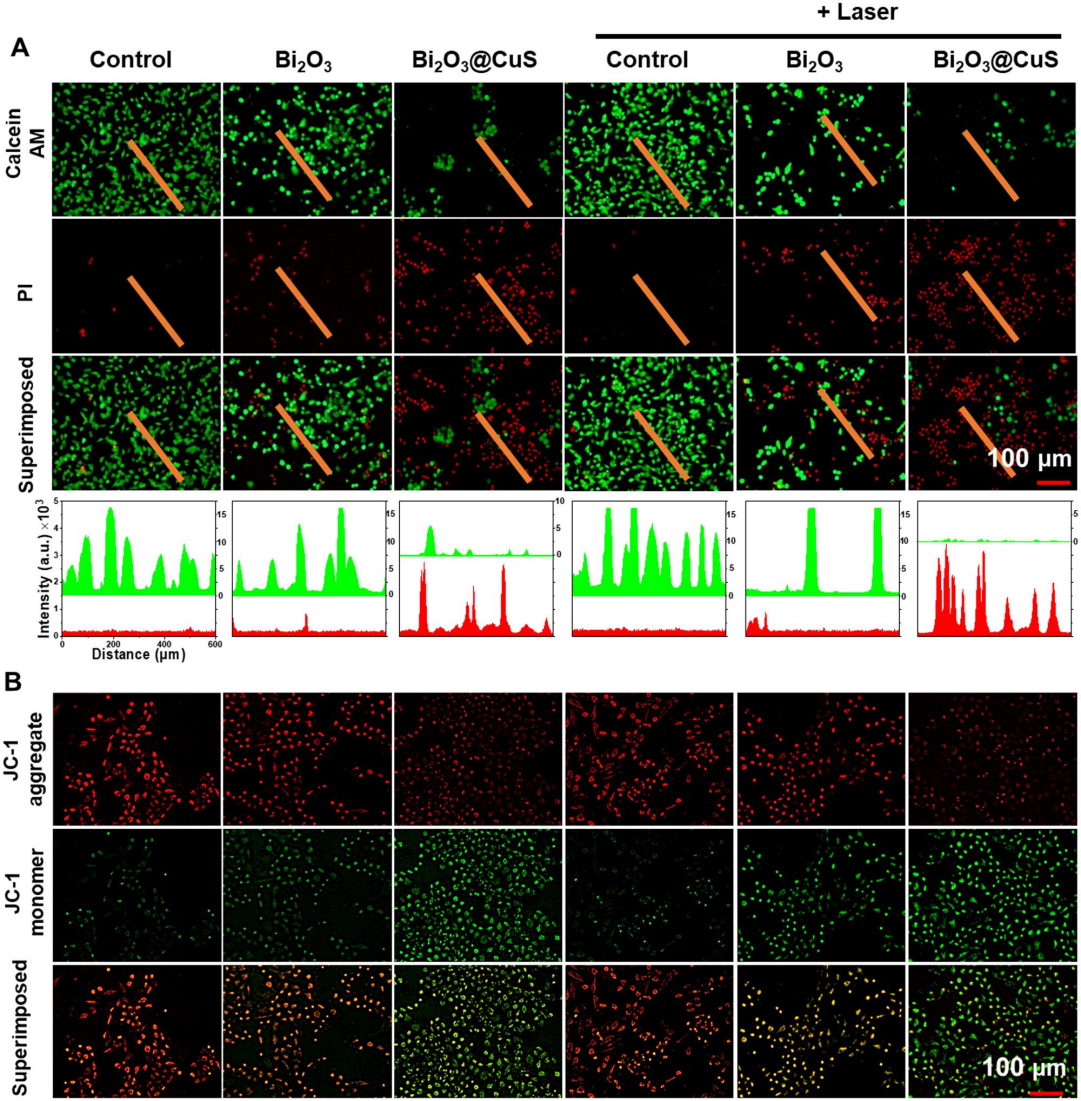
Apoptosis of 4T1 cells under different treatment conditions. (**A**) Calcein AM/PI staining indicates live and dead cells and (**B**) JC-1 staining illustrates the mitochondrial damage of Bi_2_O_3_@CuS composites intracellularly (808 nm laser irradiation in each group, time: 6 min, power: 1 W·cm^−2^).

Prior to exploring therapeutic efficacy, the biosafety of Bi_2_O_3_ and Bi_2_O_3_@CuS nanoarchitectures at the cellular level was investigated in normal fibroblasts (L929 cell line). The CCK-8 method was employed to determine the cytotoxicity of normal fibroblasts co-cultured with different concentrations of Bi_2_O_3_ and Bi_2_O_3_@CuS nanoarchitectures for 24 h. As depicted in Figure 5C, the survival rate of L929 cells co-cultured with Bi_2_O_3_@CuS nanoarchitectures at the highest concentration of 200 μg·ml^−1^ was over 80%, referring to the level-I toxicity range and exhibiting excellent biocompatibility. Further, the cytotoxicity of Bi_2_O_3_ and Bi_2_O_3_@CuS nanostructures on cancer cells (4T1 cell line) was determined by designing the same experimental conditions. The survival rate of 4T1 cells co-incubated with Bi_2_O_3_@CuS composites decreased to 40%, indicating the Fenton-like CDT effect of the CuS layer and Bi^3+^-induced oxidative stress. Interestingly, Bi_2_O_3_@CuS+Laser group at the concentration of 200 μg/ml resulted in over 90% of cell death (Figure 5D), indicating that Bi_2_O_3_@CuS nanoarchitectures could improve the cell ablating effect due to the synergistic PTT/CDT effects. Furthermore, the apoptosis of breast cancer cells was determined by live-dead staining assay by capturing the fluorescence images after co-staining the cells with the Calcein AM/PI dye mixture. Compared with the control groups in the corresponding dark and light irradiation circumstances, the Bi_2_O_3_@CuS and Bi_2_O_3_@CuS+Laser groups showed significantly more cells in red color, indicating irreversible damage by the designed composites (Figure 6A). In addition, the ablating intensity of the Bi_2_O_3_@CuS+Laser group in tumor cells was higher than that of the only Bi_2_O_3_@CuS group in the dark, demonstrating the synergistic CDT and PTT effects causing more dreadful. To further validate the cellular apoptosis effect, the intracellular mitochondrial damage was determined by employing the JC-1 staining kit, as the changes in the mitochondrial membrane potential (MMP) levels could indicate substantial cellular apoptosis. As shown in Figure 6B, bright red fluorescence was observed in the Control, Laser and Bi_2_O_3_ groups, implying that the changes in the MMP levels could indicate that cells were alive and had no severe toxicity. Contrarily, Bi_2_O_3_@CuS and Bi_2_O_3_@CuS+Laser treatment groups resulted in gradually increased green fluorescence, representing severe damage to the intracellular mitochondria. Altogether, Bi_2_O_3_@CuS composites exhibited synergistic therapeutic effects, exploring the mitochondrial damage and subsequent apoptosis of 4T1 cells.

#### ROS generation

As confirmed by the CDT effect *in vitro* (as stated in CDT efficacy), the intracellular ROS generation, i.e. •OH radicals, mediated by Bi_2_O_3_@CuS nanoarchitectures in the presence of H_2_O_2,_ was validated. DCFH-DA was employed as the ROS probe, which could be oxidized by intracellularly generated ROS to green-colored DCF. Simultaneously, the reduced GSH assay kit was applied to evaluate the intracellular GSH level after DMEM, Bi_2_O_3_ and Bi_2_O_3_@CuS treatments. The intracellular GSH levels after Bi_2_O_3_@CuS treatment were lower than other groups, indicating that they could consume intracellular GSH and convert Cu^2+^ to Cu^+^, facilitating further Fenton-like reactions. In addition, Bi_2_O_3_ constructs played the role of GSH depletion in tumor cells (Figure 5E), greatly reducing the intracellular GSH content and indicating the GSH depletion toward improved CDT effect. As depicted in Figure S8, no DCF fluorescence indicating ROS was observed in the blank treatment group of 4T1 cells. In contrast, the positive control group showed brighter green fluorescence than the blank control group. Contrarily, the Bi_2_O_3_@CuS group showed higher levels of green fluorescence compared with the control treatment group, indicating the CDT effect. Under NIR laser irradiation, the intensity of green fluorescence further increased in the Bi_2_O_3_@CuS group, which might be caused by the PTT effect, indicating a synergistic CDT-PTT effect.

### *In vivo* investigations

#### CT imaging

**Figure 7.**
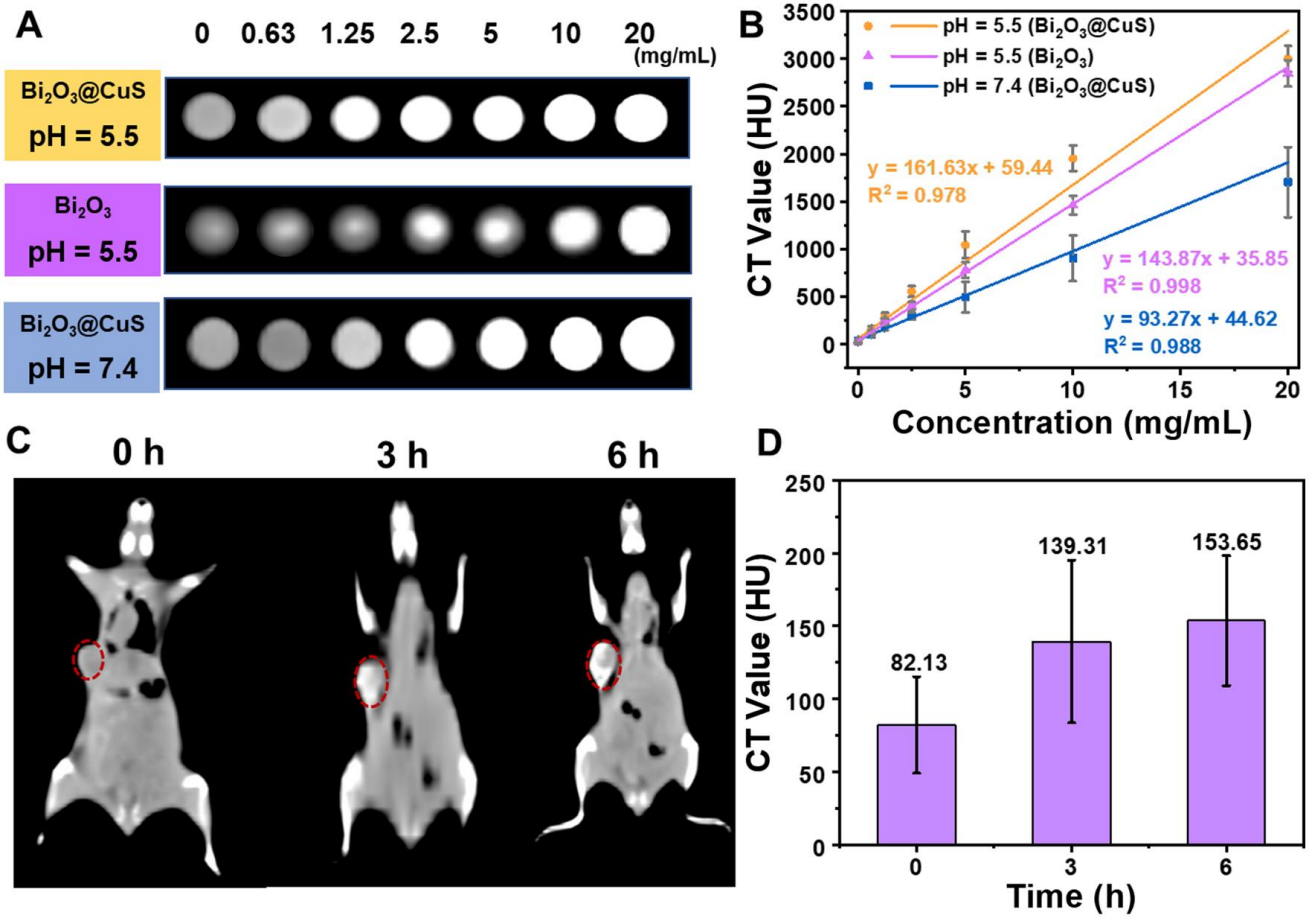
CT Imaging efficacy in vitro and in vivo. (**A**) CT imaging effects of different concentrations of Bi_2_O_3_ and Bi_2_O_3_@CuS at various pH levels and (**B**) corresponding HU values (unit: ml·mg^−1^). (**C**) CT images of the tumor site in 4T1 tumor-bearing mice before administration 0, 3 and 6 h after intravenous injection of Bi_2_O_3_@CuS (5 mg·ml^−1^, 100 μl). (**D**) The average intensity of CT signals retrieved from the tumor site in (**C**).

Considering the high atomic number of Bi (*Z* = 83), Bi-based materials are used as CT contrast agents, offering excellent radiosensitizer effect [76]. To demonstrate the role of CuS on the contrast efficacy of Bi_2_O_3_, the designed nanoparticles were initially exposed at different concentrations in solutions of altered pH (5.5 and 7.4) values. As shown in Figure 7A, both Bi_2_O_3_ and Bi_2_O_3_@CuS offered exceptional CT imaging effects under different pH environments at high concentrations. Nevertheless, Bi_2_O_3_@CuS at the low concentration of 1.25 mg·ml^−1^ presented remarkable CT contrast effects under acidic conditions over the physiological environment at a similar concentration. Specifically, Bi_2_O_3_@CuS at the low concentration of 1.25 mg·ml^−1^ presented remarkable CT contrast effects under acidic conditions over the physiological environment at a similar concentration. The improvement in the CT imaging of the designed nanoarchitectures might be due to the degradation of the outer CuS layer in the mild acidic TME. After acid-responsive CuS degradation, the resultant Bi^3+^ and Cu^2+^ species might enhance the ability to absorption of X-rays [59]. Accordingly, the degradation degree of Bi_2_O_3_ and CuS was greatly increased with the increased concentration of the nanoformulation, substantially improving the content of the two metal ions. By linearly fitting the CT values at different concentrations under different pH environments, the HU slopes of Bi_2_O_3_@CuS at pH—5.5 (tumor-mimic) and pH—7.4 (blood-mimic) were 161.63 and 93.27 ml·mg^−1^, respectively. In contrast, the HU slope of pristine Bi_2_O_3_ at pH—5.5 was 143.87 ml·mg^−1^, which was lower than Bi_2_O_3_@CuS at pH—5.5, indicating CuS nanoplating improved CT imaging efficacy of Bi_2_O_3_ (Figure 7B). Considering the optimal feeding ratio and other synthesis conditions, the designed Bi_2_O_3_@CuS nanoarchitectures at a Cu: Bi ratio of 1:1 showed no influence on the CT contrast efficacy. Although the CuS facilitated the insignificant rise in the HU slope value of Bi_2_O_3_ in the tumor-mimicking environment, the HU values obtained by fitting were significantly higher than the CT values reported in existing literature, such as BiPt-PFA (50.16 HU ml·mg^−1^) [77], Bi-PEG NCs (60.3 HU ml·mg^−1^) [78] and Bi-BSA@DOX (66.7 HU ml·mg^−1^) [79].

The *in vivo* CT imaging was evaluated after intravenous injection of Bi_2_O_3_@CuS nanoarchitectures using 4T1 bearing tumors. Before (0 h) injection of Bi_2_O_3_@CuS nanoarchitectures, only normal bone structures were visible (Figure 7C). Contrarily, strong CT imaging signals appeared at the tumor site after (3 h) injection of Bi_2_O_3_@CuS. With an increase in the exposure period, the CT imaging signal of the mouse tumor site showed a significant increase in the contrast efficacy after 6 h. Simultaneously, the quantitative analysis was conducted based on the CT values of mouse tumor sites at different periods (Figure 7D), explicitly indicating the time-dependent CT contrast efficacy. Although there was a slight increase in CT efficacy, the animal images indicated more nanoarchitectures at the tumor site in 6 h over 3 h, greatly enriching the CT imaging effect. These findings of CT efficacy, both *in vitro* and *in vivo,* could interpret the crucial roles of the designed Bi_2_O_3_@CuS nanoarchitectures in real-time monitoring and guidance during the treatment of tumors as a potential CT contrast agent in clinical medicine.

#### Anti-tumor efficacy

**Figure 8.**
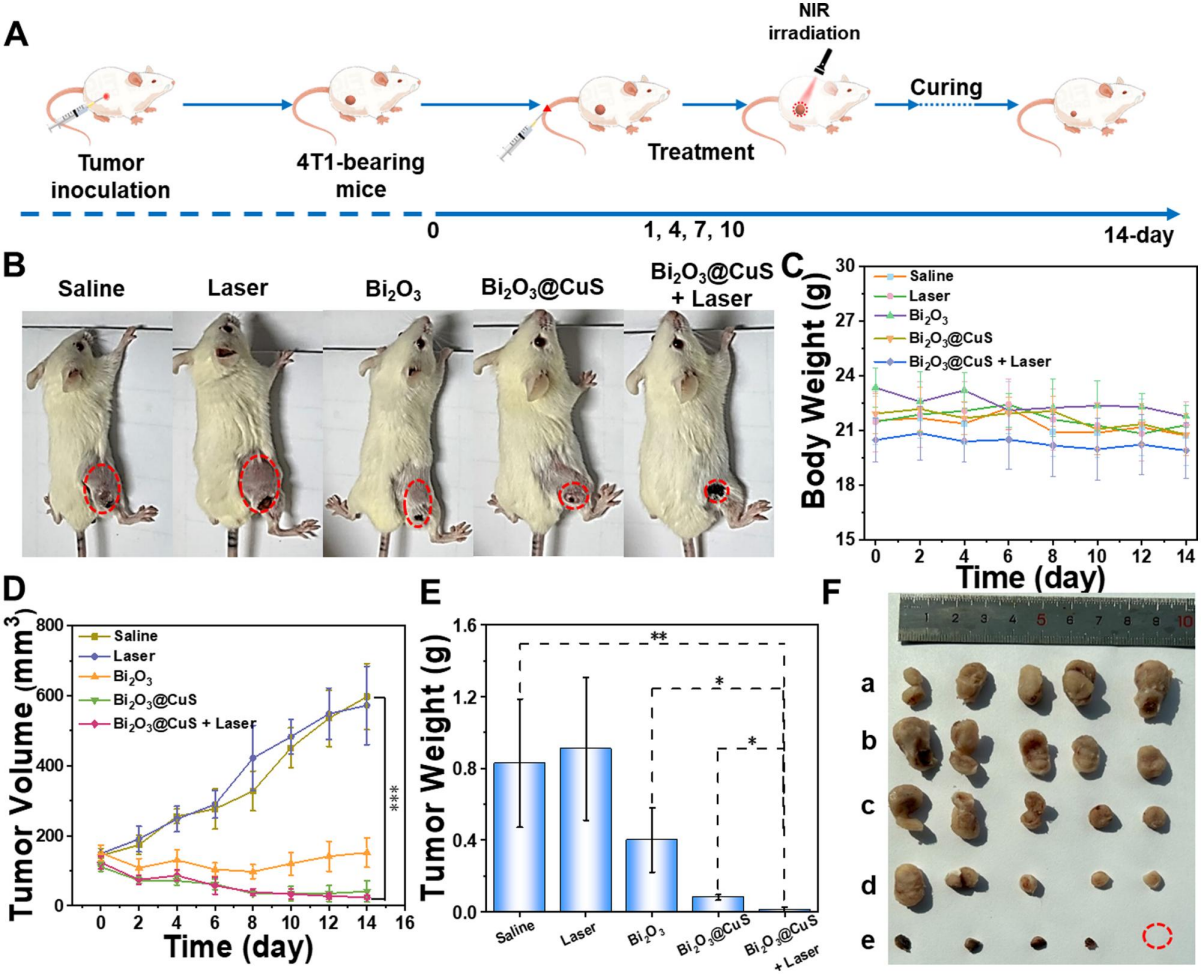
Evaluation of tumor inhibition effect in mice in vivo. (**A**) Schematic diagram of mouse treatment flow. Tumor-bearing mice in different treatment groups. (**B**) Representative pictures of mice after the end of treatment, the tumor site is circled, the change curve of (**C**) body weight, (**D**) tumor volume curve, (**E**) tumor weight change diagram and (**F**) tumor anatomy photos during the treatment. Among them, (a–e) samples correspond to figure (**D**). The laser samples were irradiated with 808 nm laser irradiation (1 W·cm^−2^) in each group for 6 min. * represents *P* < 0.05 and ** indicates *P* < 0.01.

Accordingly, the designed Bi_2_O_3_@CuS nanoarchitectures were subjected to the 14-day treatment in tumor-bearing mice (Figure 8A) by setting up five experimental treatment groups. Figure 8B shows the bright-field view images of mice on the last day after treatment. It was observed that Bi_2_O_3_@CuS and Bi_2_O_3_@CuS+Laser treatment groups of tumor-bearing mice showed substantially smaller tumors than others. In addition, the body weight of mice in each group showed no obvious change, indicating compatibility with no abnormal changes in the body weight (Figure 8C). Compared with the other treatment groups, the Bi_2_O_3_@CuS and Bi_2_O_3_@CuS+Laser treatment groups showed better inhibition efficacy. Simultaneously, the latter group exhibited improved therapeutic effects, which could be due to the light-induced PTT effect, as well as the Fenton-like reactivity-based CDT effect synergistically (Figure 8D–F). Accordingly, the tumor volume of Bi_2_O_3_@CuS and Bi_2_O_3_@CuS+Laser treatment groups demonstrated a significant reduction in tumor growth with time (Figure 8D). Although the difference in tumor volume after treatment between the last two groups of mice in Figure 8D was insignificant, the Bi_2_O_3_@CuS+Laser treatment group eventually showed no signs of the tumor with negligible residual tumor weights due to PTT/CDT synergistic treatment (Figure 8E). As depicted in Figure 8F, the visual images of the tumor explained that the tumors of the Bi_2_O_3_@CuS+Laser treatment group of mice showed a complete recovery without recurrence. In addition, the small difference in tumor volume could be due to the crusting of the skin after the photothermal treatment. These findings indicated excellent PTT and CDT effects toward ablating breast carcinoma *in vivo*.

#### Biocompatibility and metabolism

Eventually, we systematically evaluated the biological safety of Bi_2_O_3_@CuS nanoarchitectures *in vivo* from multiple perspectives, such as hemolysis assay, H&E staining and serological analyses. The hemolysis rate of different nanoarchitectures (Bi_2_O_3_ and Bi_2_O_3_@CuS nanoarchitectures) obtained from the fresh blood samples of mice was less than 5% till the concentration of 200 μg·ml^−1^ (Figure S9A and B). However, the Bi_2_O_3_@CuS sample at a concentration of 400 μg·ml^−1^ resulted in a slightly higher hemolysis rate of about 10% over the concentration of 200 μg·ml^−1^. The reasons could be the high concentration, leading to the increased difference between internal and external pressure and substantial hemolysis rate.

**Figure 9.**
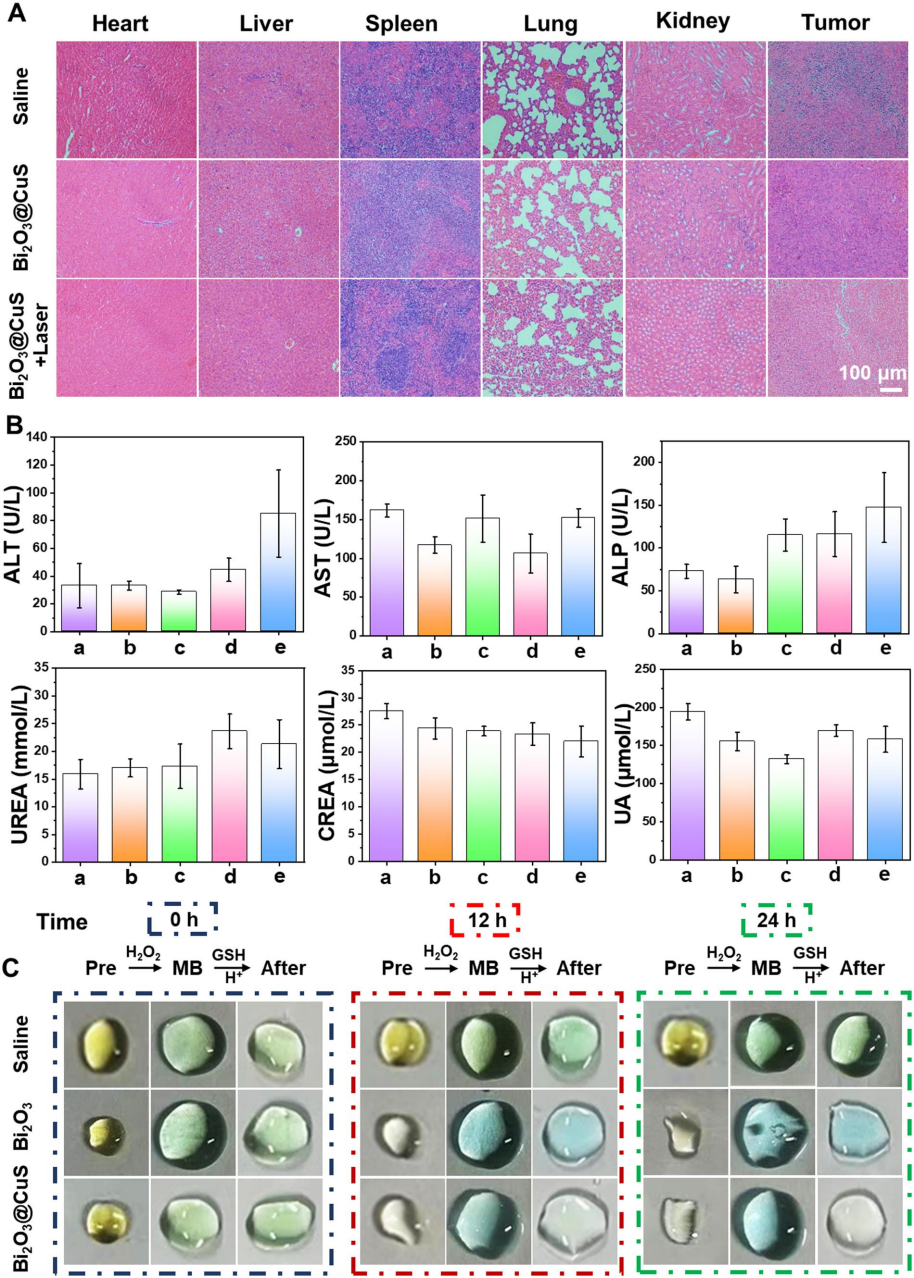
Biosafety and metabolism evaluations of Bi_2_O_3_@CuS. (**A**) H&E staining of various organs of tumor-bearing mice in different treatment groups (scale = 100 µm). (**B**) Blood biochemical analysis of tumor-bearing mice in different treatment groups, where samples (1)–(5) refer to the saline, laser, Bi_2_O_3_, Bi_2_O_3_@CuS and Bi_2_O_3_@CuS+laser groups, respectively (808 nm laser irradiation in each group, time: 6 min, power: 1 W·cm^−2^). (**C**) Color changes of MB degradation in urine at different periods (0, 12 and 24 h).

Simultaneously, the normal mice were injected with different nanoarchitectures, and the collected blood samples from the eyeballs of mice were subjected to biochemical analysis. Accordingly, several biochemical indicators were recorded to demonstrate the functions of major organs. As shown in Figure S9C, the liver function indicators, including alanine aminotransferase, aspartate aminotransferase and alkaline phosphatase, and kidney function indicators, such as urea nitrogen (urea nitrogen) and uric acid levels remained within the normal range. These findings implied that liver and kidney functionalities were not affected by Bi_2_O_3_ and Bi_2_O_3_@CuS treatment in mice. After treatment with the designed nanoarchitectures, the histological characteristics of major organs (heart, liver, spleen, lung and kidney) and tumors were detected by H&E staining (Figure 9A). No obvious pathological morphological damage was observed in the organ sections after treatment with the designed nanoarchitectures, indicating excellent biocompatibility. Simultaneously, mice (*n* = 3) in each treatment group were randomly selected for blood biochemical and blood routine analysis. Accordingly, the blood analysis indicated that the mice treated with the designed nanoarchitectures were in the normal range, with no obvious difference compared with the control group (Figure 9B). In addition, the number of WBCs was significantly lower in the Bi_2_O_3_@CuS and Bi_2_O_3_@CuS+Laser treatment groups than in the control group, which were within the reference range of 0.8–6.8×10^9^/l. Moreover, the inflammation was significantly reduced in the Bi_2_O_3_@CuS and Bi_2_O_3_@CuS+Laser treatment groups over the control group, showing significant differences (Figure S10). The remaining treatment groups showed no obvious abnormalities. Considering these attributes from multiple perspectives, Bi_2_O_3_@CuS indicated exceptional biocompatibility in blood, organ, tissue and cellular levels, indicating their tolerance *in vivo*. Together, these designed nanoarchitectures presenting excellent biocompatibility on the cellular and animal levels could be suitable for formulating antitumor dosage forms.

Finally, the collected urine samples were analyzed to investigate the degradation efficacy of administered Bi_2_O_3_ and Bi_2_O_3_@CuS nanomedicines in tumor-bearing mice at 0, 12 and 24 h. After incubation with solutions containing MB, H_2_O_2_ and GSH in the shaker at 37°C for 12 h, the urine color of mice treated with Bi_2_O_3_@CuS changed from blue to the original urine color **(**Figure 9C**)**. The altered color after the reaction might be due to the presence of Cu^2+^ and Cu^+^ formed by Bi_2_O_3_@CuS degradation in the urine. The resultant •OH produced by Cu ions could degrade MB in the presence of both GSH and H_2_O_2_. In addition, the urine of mice in treatment with Bi_2_O_3_ became lighter after incubation, which might be due to the reaction between Bi ions in the urine and GSH, resulting in a lighter color of the solution. The change in the color of urine indicated that these materials showed exceptional excretion efficacy. Thus, we believe that these Bi_2_O_3_@CuS nanoarchitectures with exceptional excretion efficacy and biocompatibility could offer no aggregation-induced toxicity to tissues and organs, enriching the EPR effect and enhancing the excretion after therapeutic efficacy from the body.

## Conclusion

In summary, we have successfully fabricated an innovative drug-like CuS-nanoplated bismuth oxide nanocomposites by a two-step process using a hydrothermal method. After systematic characterization of physicochemical properties, the photothermal and chemodynamic effects of these nanocomposites were substantially explored *in vitro* due to their band absorption at 808 nm toward executing localized PTT effects and excellent toxic ROS levels intracellularly in the TME, respectively. Considering synergistic PTT/CDT effects, the nanoplated CuS on the surface would decompose precisely in the TME, exposing the core Bi_2_O_3_ nanoparticles and facilitating GSH depletion toward tumor ablation. In addition, these pH-responsive Bi_2_O_3_ constructs could efficiently be applied as CT contrast agents to monitor the treatment process in real-time and track its metabolism through the kidney and liver *in vivo*. Finally, we systematically validated the biosafety of the designed composites at the blood, cell, tissue and organ levels. Together, these designed pH-/NIR-responsive Bi_2_O_3_@CuS nanoarchitectures could achieve exceptional PTT/CDT synergy, greatly enhancing the therapeutic effect against TNBC. Simultaneously, the high atomic number of Bi could further achieve CT imaging effects, providing real-time tracking and superior therapeutic effects against TNBC that were prone to metastasis and recurrence. Despite the optimization, it is necessary to explore the synthesis of these nanoarchitectures in large amounts and determine the casing of CuS over Bi_2_O_3_ precisely concerning the reproducibility. In addition to the assessment of biosafety at various levels, it is important to present the roles of released ions on the genomic levels. Together, further investigations are required to explore the possibility of application prospects of designed Bi_2_O_3_@CuS nanoarchitectures in pharmaceutics.

## Supplementary Material

rbae128_Supplementary_Data

## Supplementary data

The supporting information file cotains SEM, TEM and DLS measurements of Bi_2_O_3_ nanospheres, EDS of Bi_2_O_3_@CuS constructs, TEM images of Bi_2_O_3_@CuS at different feeding ratios (Bi_2_O_3_: CuS) and different reaction times of Cu_2_O, the Extinction coefficient of Bi_2_O_3_@CuS nanoparticles, photothermal conversion efficiency and calculated η value of Bi_2_O_3_@CuS, ^1^O_2_ generation by different treatment groups, Acid-responsive degradation of designed Bi_2_O_3_@CuS constructs, ROS and biosafety evaluation of Bi_2_O_3_@CuS and hematologic analysis of homozygous mice in different Bi_2_O_3_@CuS-based treatment groups.
