# Correction to: Nanoarchitectonics of copper sulfide nanoplating for improvement of computed tomography efficacy of bismuth oxide constructs toward drugless theranostics

**DOI:** 10.1093/rb/rbag184

**Published:** 2026-09-16

This is a correction to: Ruo-Yin Meng, Hong-Ying Xia, Ying Zhao, Ying-Tong Ye, Shi-Bin Wang, Ai-Zheng Chen, Ranjith Kumar Kankala, Nanoarchitectonics of copper sulfide nanoplating for improvement of computed tomography efficacy of bismuth oxide constructs toward drugless theranostics, *Regenerative Biomaterials*, Volume 11, 2024, rbae128, https://doi.org/10.1093/rb/rbae128

In the originally published version of this manuscript, Figure 9 contained an inadvertently duplicated image. In Figure 9C, the “Pre” treatment image for the Saline group at the 24 h time point was unexpectedly repeated with the “Pre” treatment of the Bi_2_O_3_@CuS group at the 0 h time point. This error was unintentional and resulted from mishandling the images, leading to the reuse of two similar images.

The authors apologise for the error. The corrected Figure 9 is provided below:

**Figure:**
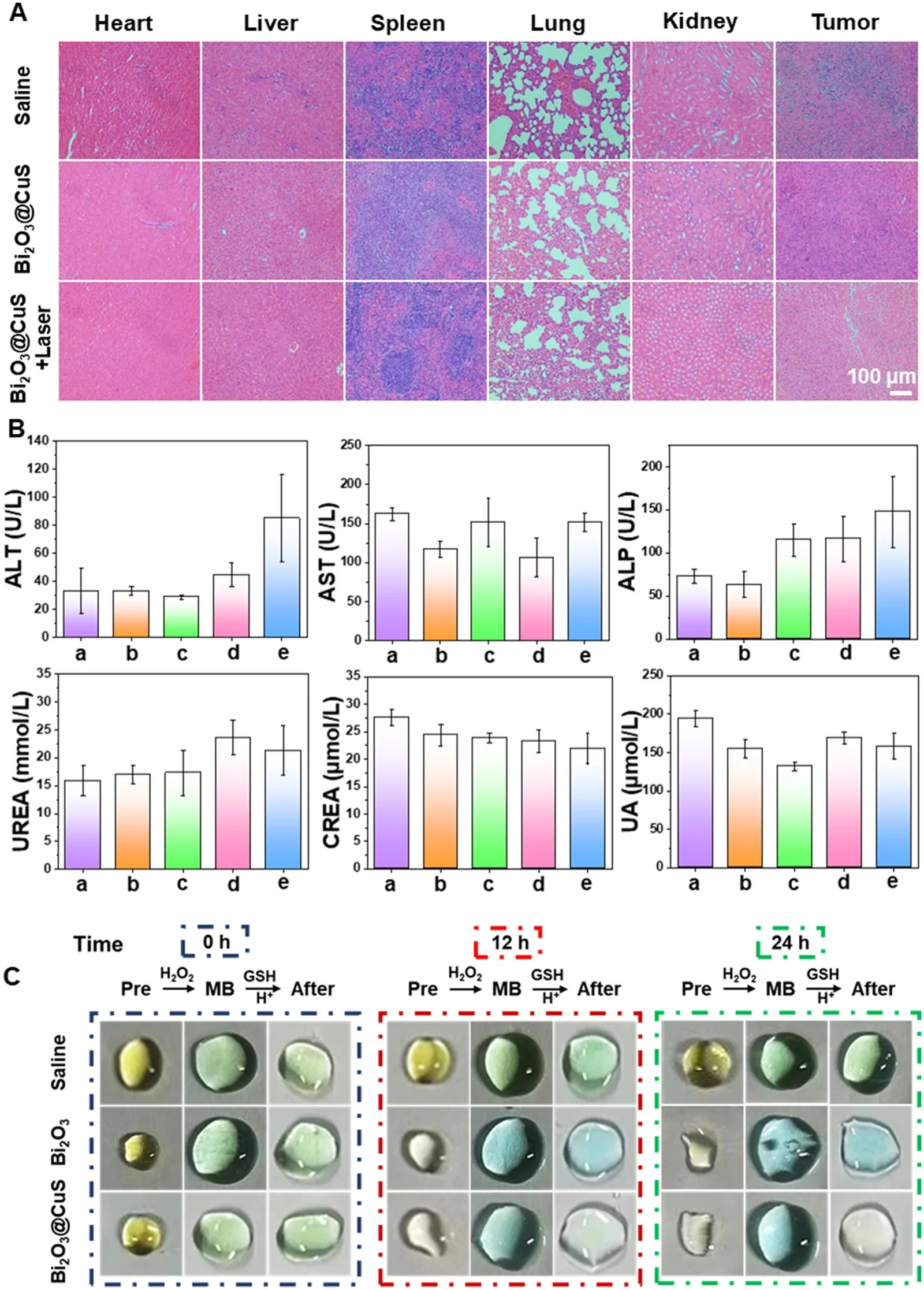


These details have been corrected only in this correction notice to preserve the version of record.

