# Supplementary material for: Nanoarchitectonics of copper sulfide nanoplating for improvement of computed tomography efficacy of bismuth oxide constructs toward drugless theranostics
Source: Regen Biomater. 2024 Oct 26;11:rbae128. doi: 10.1093/rb/rbae128 (PMC11593496; doi:10.1093/rb/rbae128)
Supplement: rbae128_Supplementary_Data [file rbae128_supplementary_data.docx]

*Supporting information*

**Nanoarchitectonics of Copper Sulfide Nanoplating for Improvement of Computed Tomography Efficacy of Bismuth Oxide Constructs towards Drugless Theranostics**

*Ruo-Yin Meng^1,2^, Hong-Ying Xia^1,2^, Ying Zhao^1,2^, Yingtong Ye^1,2^, Shi-Bin Wang^1,2,3^, Ai-Zheng Chen^1,2,3^, Ranjith Kumar Kankala^1,2,3,^**

^1^Institute of Biomaterials and Tissue Engineering, Huaqiao University, Xiamen 361021, PR China

^2^College of Chemical Engineering, Huaqiao University, Xiamen 361021, PR China

^3^Fujian Provincial Key Laboratory of Biochemical Technology (Huaqiao University), Xiamen 361021, PR China

**Address for correspondence to**:

***Email: [ranjithkankala@hqu.edu.cn](mailto:ranjithkankala@hqu.edu.cn) (R. K. K.)

ORCID: 0000-0003-4081-9179

Tel./fax: +86 592 616 2326


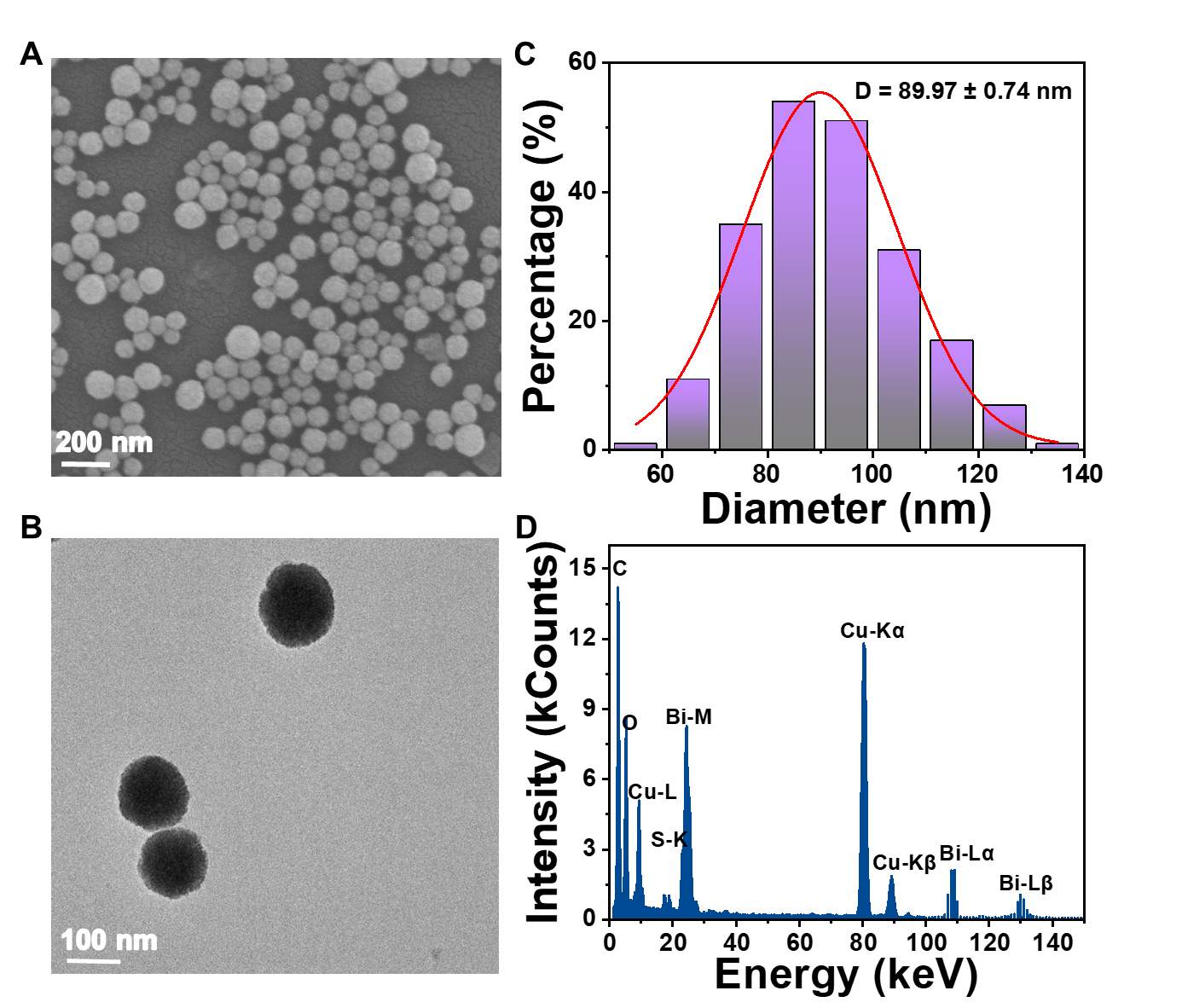


**Figure S1. Morphology and particle size characterization of Bi_2_O_3_ nanospheres.** ***(A)*** *SEM image,* ***(B)*** *TME image, and* ***(C)*** *statistical analysis of particle size of Bi_2_O_3_ nanospheres.* ***(D)*** *Elemental distribution of Bi_2_O_3_@CuS nanoarchitectures.*


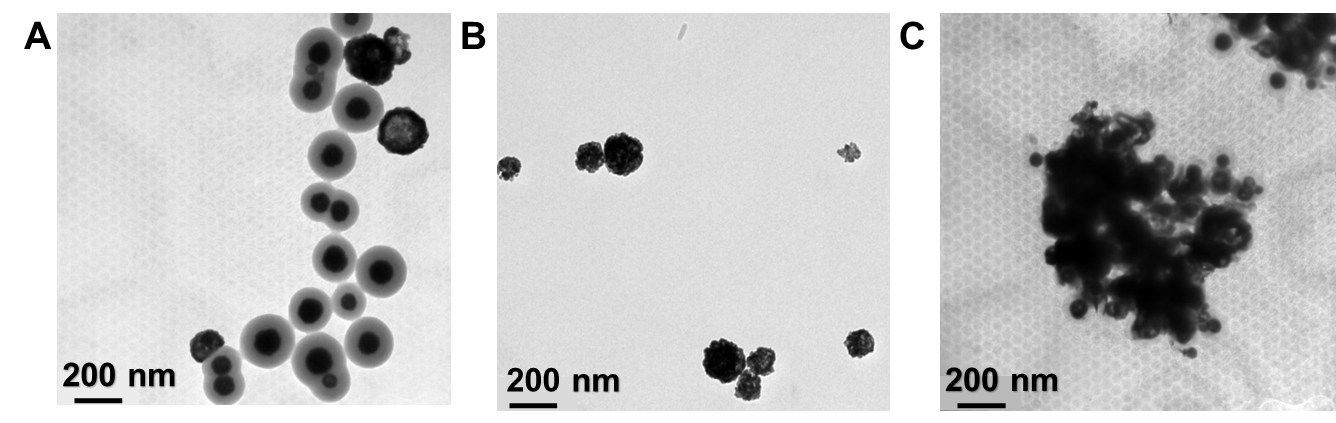


**Figure S2.** **Different reduction reaction times for Cu_2_O.** *TEM images of Bi_2_O_3_@CuS nanoarchitectures synthesized for* ***(A)*** *2 h,* ***(B)*** *4 h, and* ***(C)*** *12 h.*


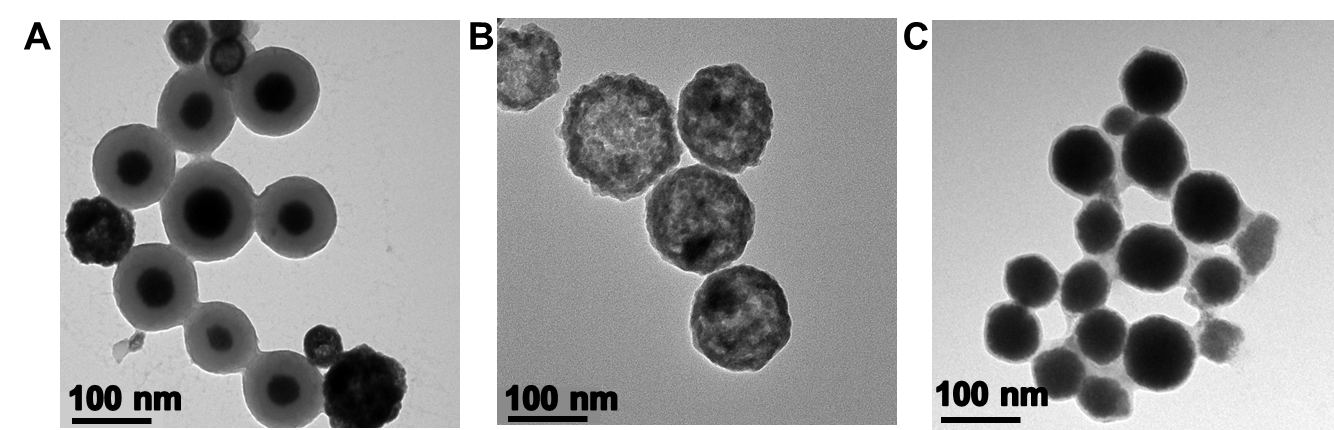


**Figure S3. The synthesis process of Bi_2_O_3_@CuS nanoarchitectures with different feed ratios for optimization of CuS nanoplating over Bi_2_O_3_**. *TEM images of Bi_2_O_3_@CuS nanoarchitectures with feed ration (Bi_2_O_3_:CuS) of* ***(A)*** *2:1,* ***(B)*** *1:2, and* ***(C)*** *1:1.*


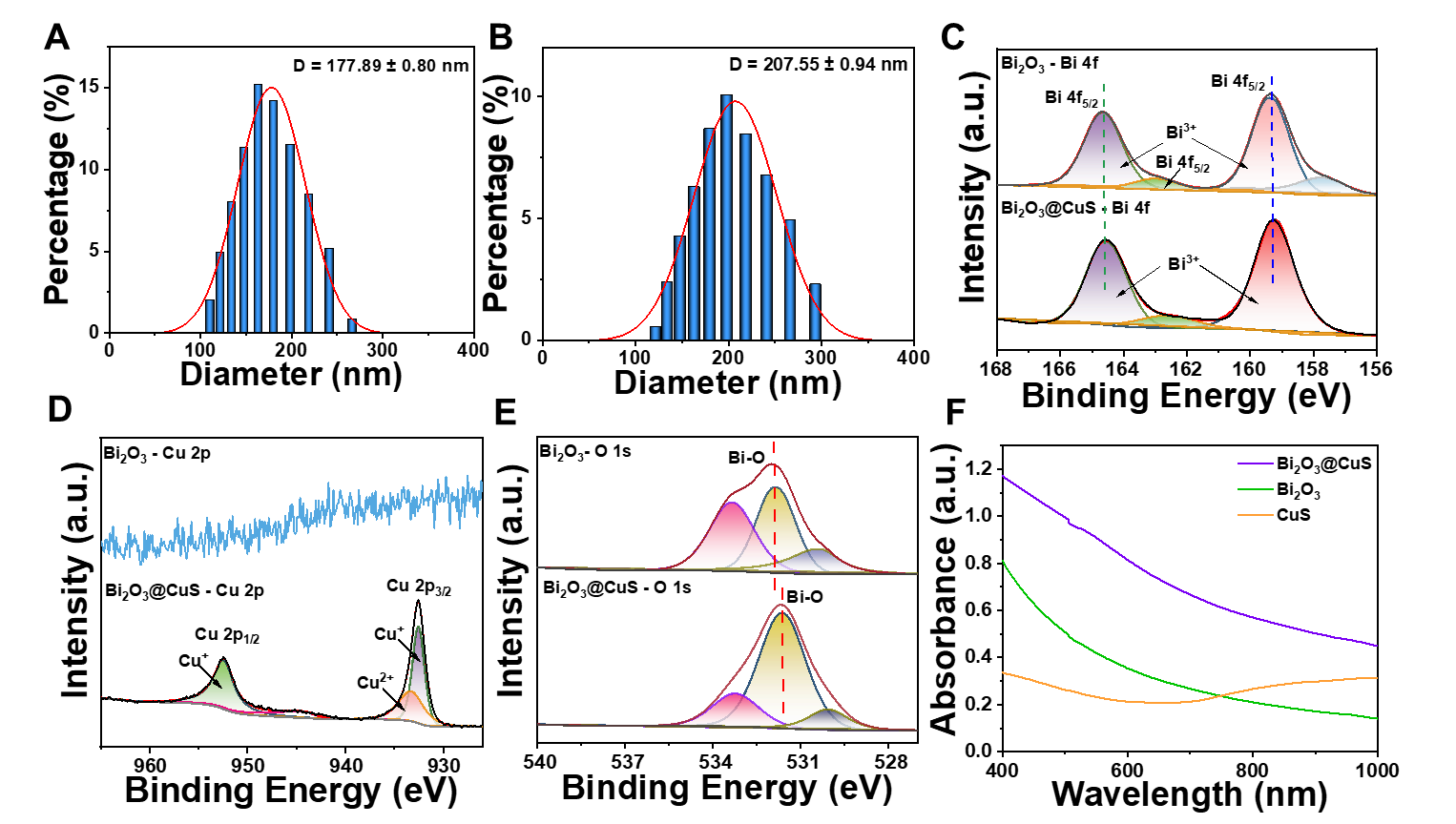


**Figure S4.** **Physicochemical characterization of Bi_2_O_3_ and Bi_2_O_3_@CuS composites.** *DLS-based particle size distribution of* ***(A)*** *Bi_2_O_3_ and* ***(B)*** *Bi_2_O_3_@CuS nanoarchitectures. High resolution* ***(C)*** *Bi 4f spectrum,* ***(D)*** *Cu 2p spectrum, and* ***(E)*** *O 1s spectrum of Bi_2_O_3_ and Bi_2_O_3_@CuS.* ***(F)*** *UV-vis absorption spectra of Bi_2_O_3_, CuS, and Bi_2_O_3_@CuS nanoarchitectures.*


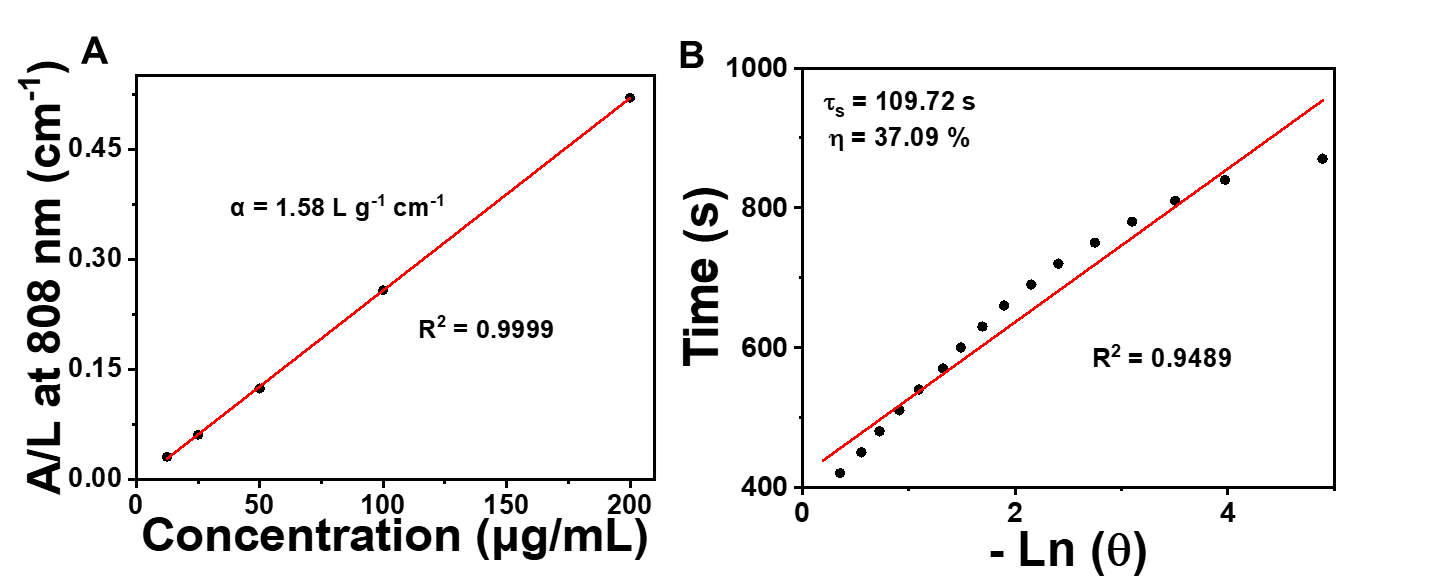


**Figure S5. Different coefficients from linear fitting.** ***(A)*** *Mass extinction coefficients of the fitted UV-absorption at 808 nm for Bi_2_O_3_@CuS solutions of various concentrations.* ***(B)*** *Time constants (τ_s_) obtained by photothermal cycle fitting and calculated photothermal conversion efficiencies (η). (808 nm laser irradiation at a power of 1* *W·cm^-2^ for 6 min).*


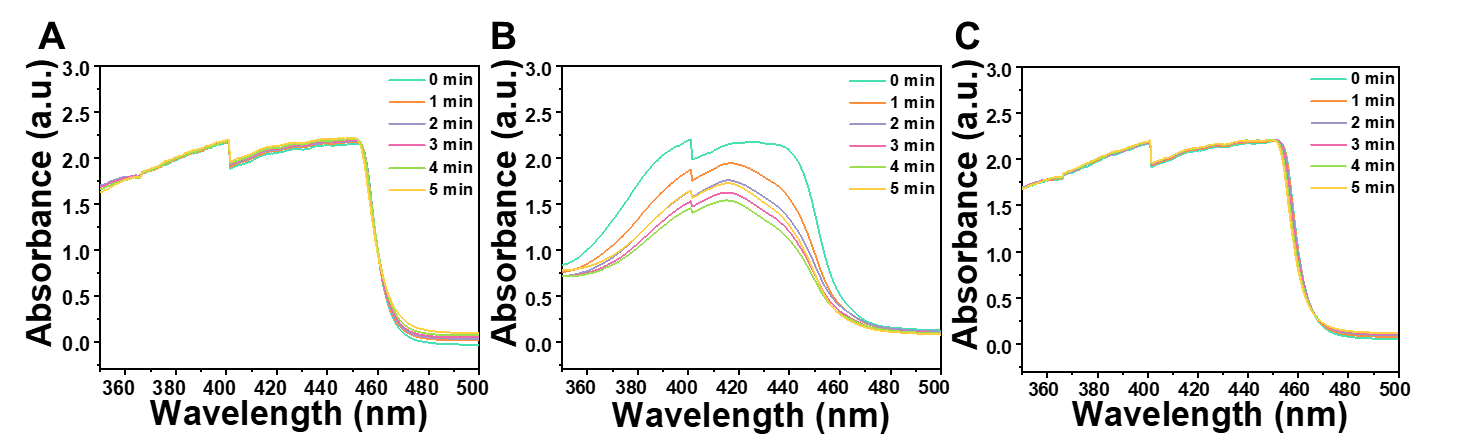


**Figure S6.** **Single-linear state oxygen generation.** *UV-vis absorption spectra of* ***(A)*** *Water,* ***(B)*** *IR780, and* ***(C)*** *Bi_2_O_3_ at different periods of DTNB irradiated with 808 nm laser (1* *W·cm^-2^).*


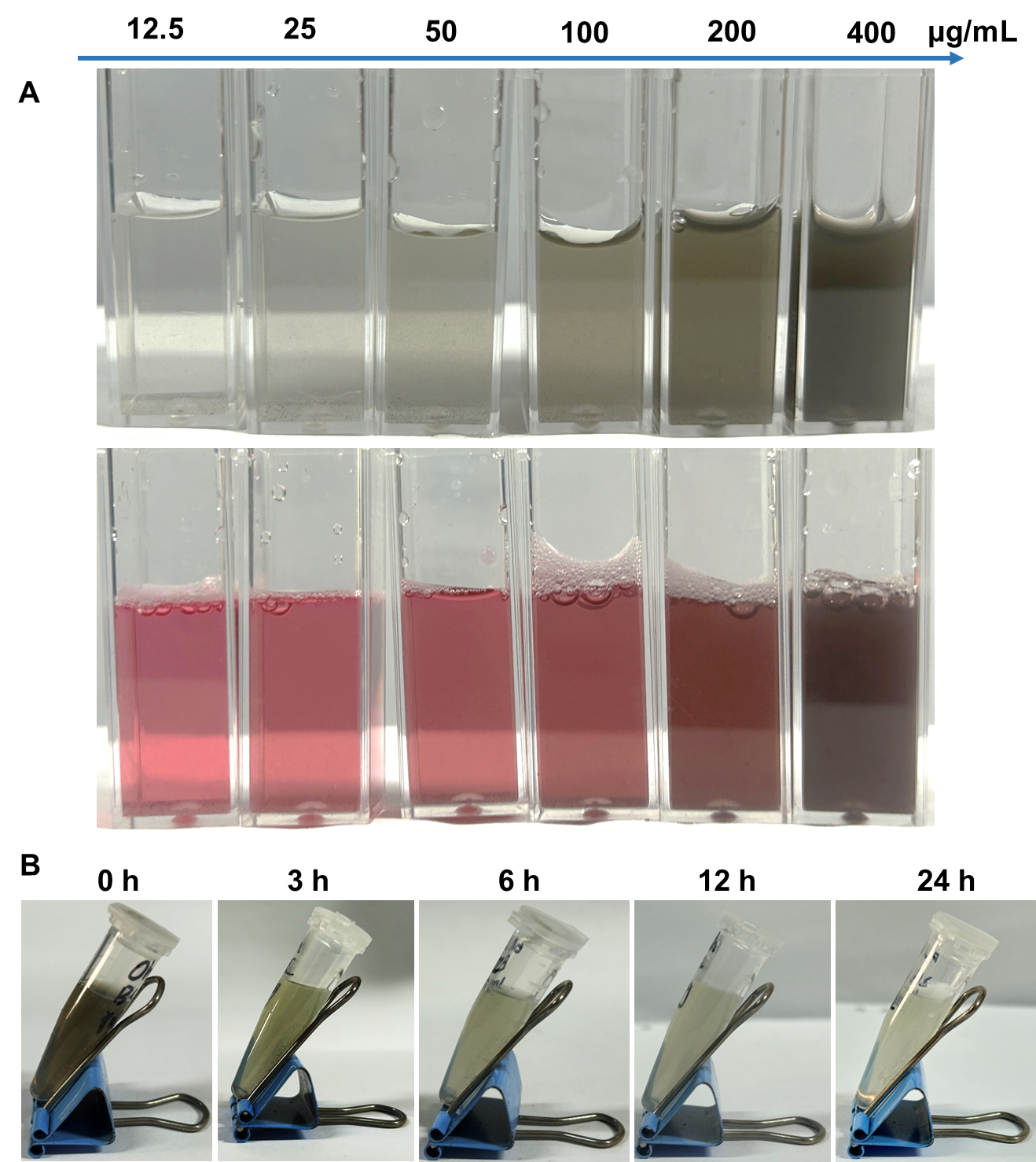


**Figure S7.** **Investigation of Suspension and Degradation. *(A)*** *Solution images of different concentrations in the aqueous phase and FBS-containing DMEM.* ***(B)*** *Digital images illustrating the degradation of Bi_2_O_3_@CuS after various time intervals in the acidic environment containing GSH (0, 3, 6, 12, and 24 h).*


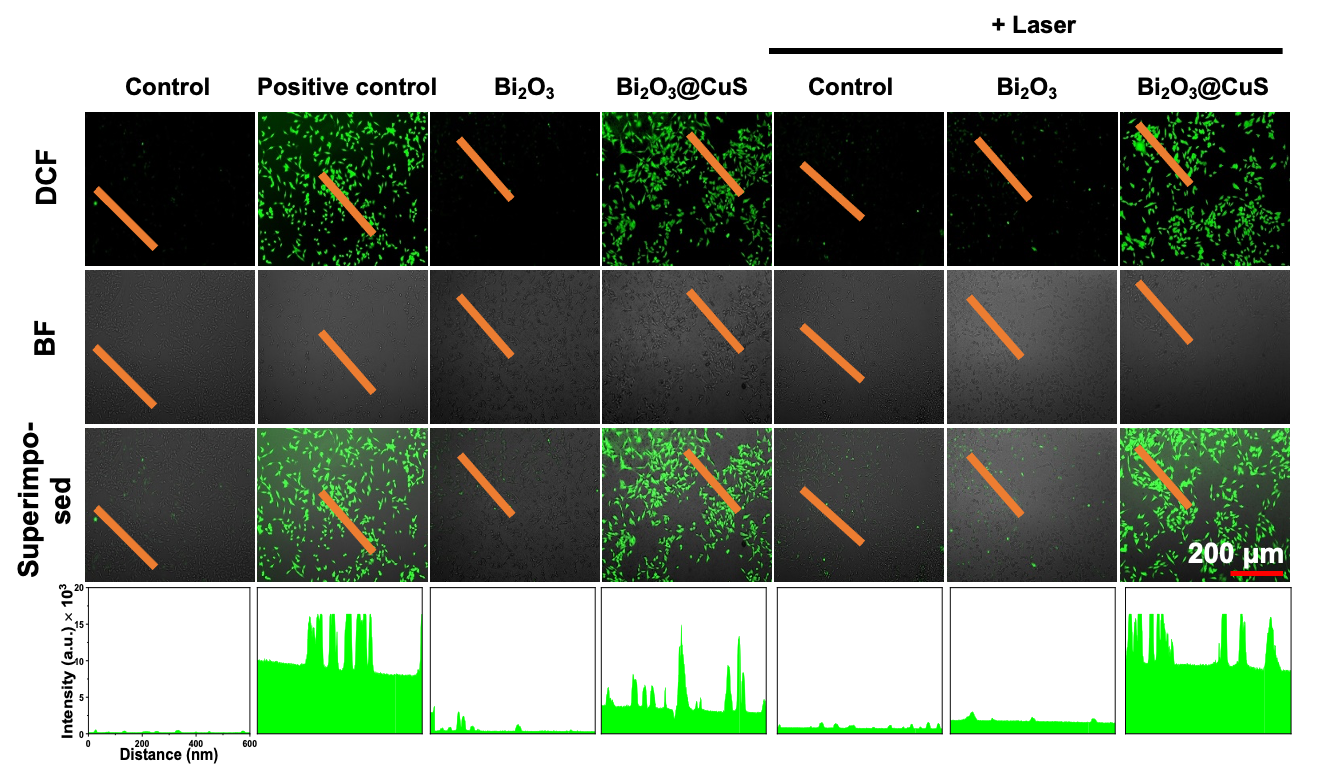


**Figure S8. Intracellular ROS generation under different conditions.** *CLSM images illustrate DCF fluorescence that correlates to the ROS levels in 4T1 cells treated with different samples (808 nm laser irradiation in each group, time: 6 min, power: 1* *W·cm^-2^).*


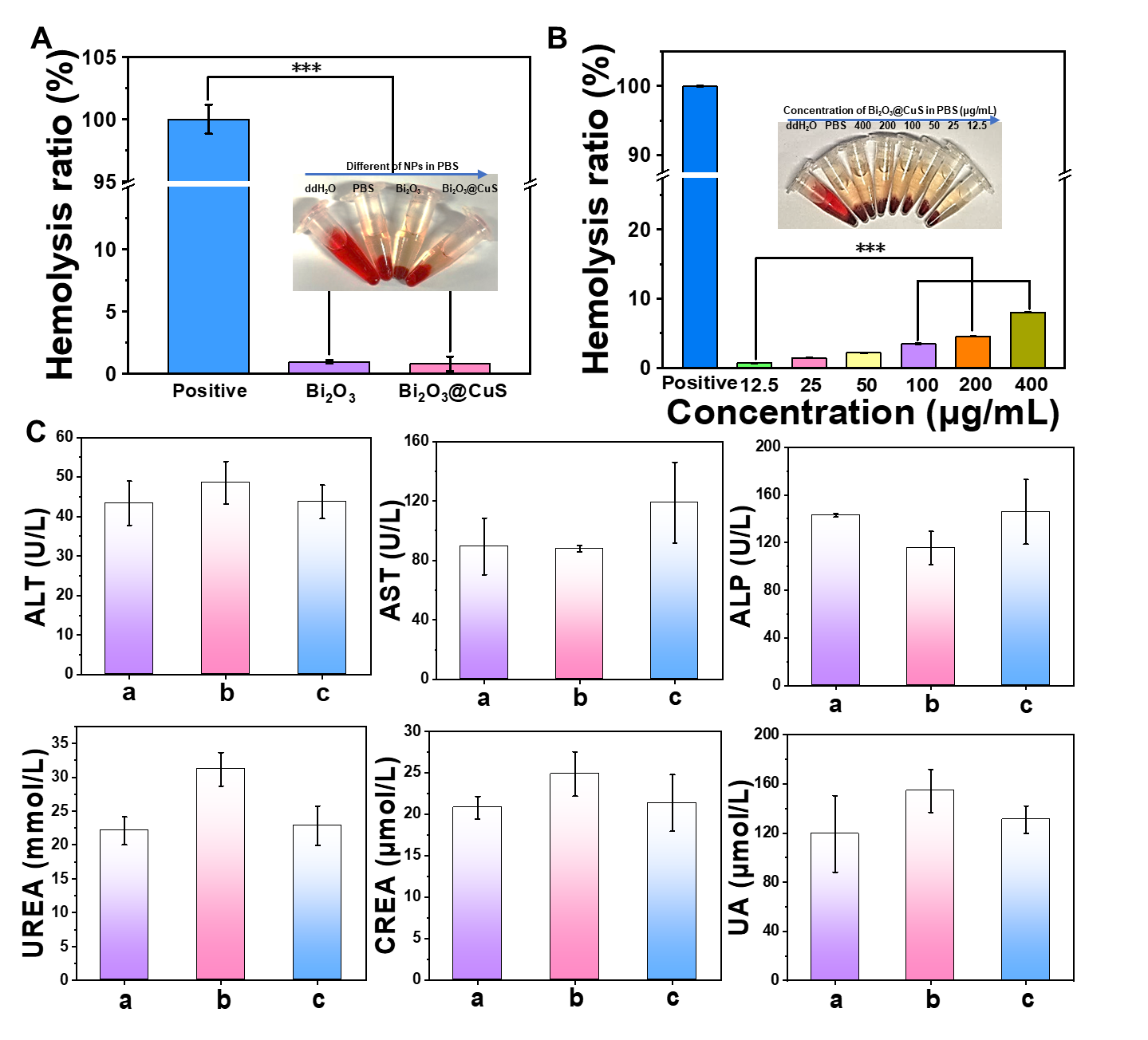


**Figure S9. Biosafety evaluation of Bi_2_O_3_@CuS.** ***(A)*** *Hemolysis rate of different nanoparticles and* ***(B)*** *different Bi_2_O_3_@CuS concentrations.* ***(C)*** *Blood biochemical analysis of normal mice in various treatment groups. Untreated mice served as the control group. Where (a - c) refer to the saline, Bi_2_O_3,_ and Bi_2_O_3_@CuS groups, respectively. (808 nm laser irradiation in each group, time: 6 min, power: 1* *W·cm^-2^).*


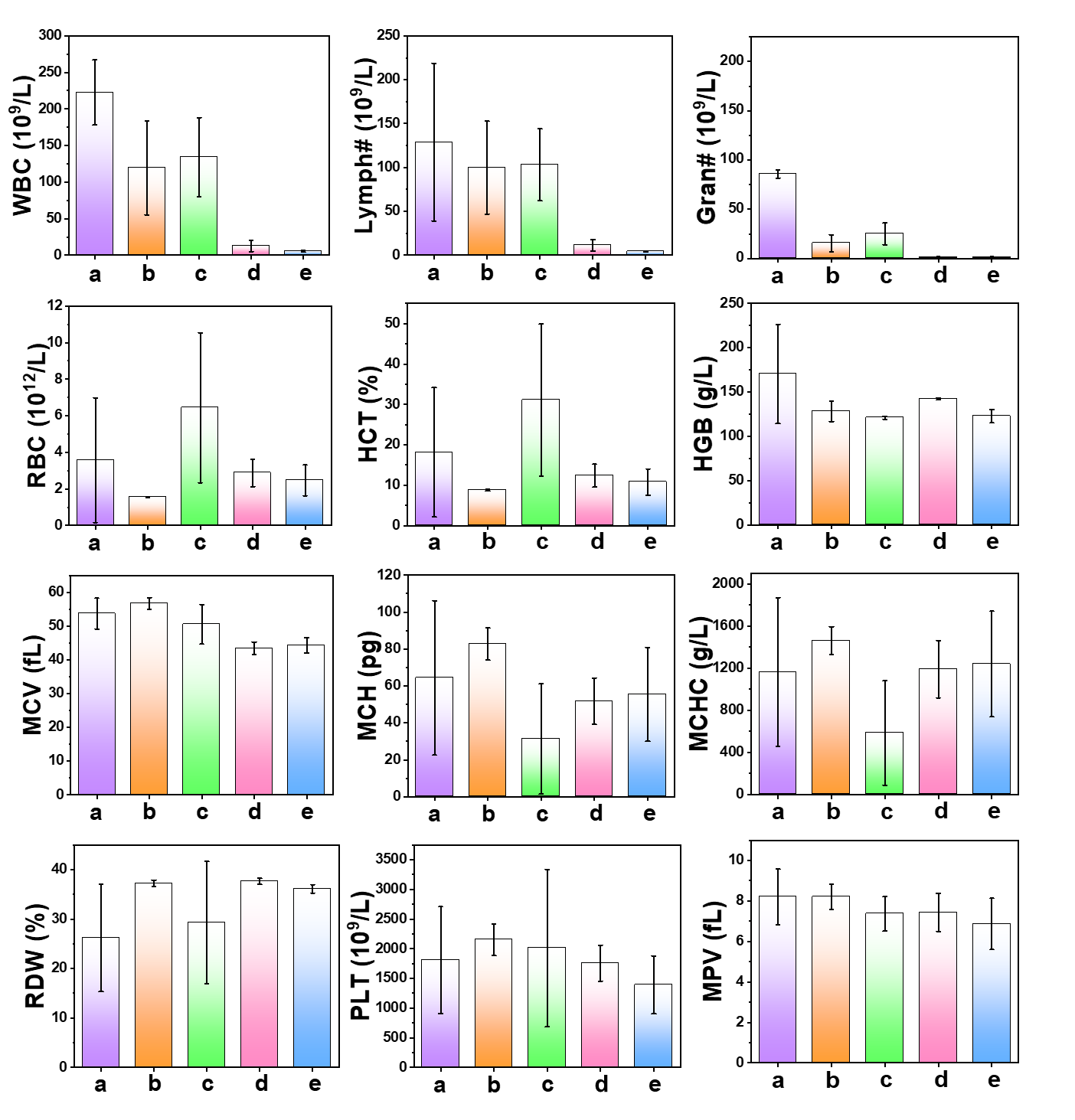


**Figure S10. Biosafety evaluation of Bi_2_O_3_@CuS.** *Routine blood analysis of tumor-bearing mice in different treatment groups,* *where (a - e) refer to the saline, Laser, Bi_2_O_3_, Bi_2_O_3_@CuS, and Bi_2_O_3_@CuS + Laser groups, respectively. (808 nm laser irradiation in each group, time: 6 min, power: 1* *W·cm^-2^).*
